# LncRNA ELF3-AS1 inhibits gastric cancer by forming a negative feedback loop with SNAI2 and regulates ELF3 mRNA stability via interacting with ILF2/ILF3 complex

**DOI:** 10.1186/s13046-022-02541-9

**Published:** 2022-12-02

**Authors:** Dandan Li, Li Shen, Xudong Zhang, Zhen Chen, Pan Huang, Congcong Huang, Shanshan Qin

**Affiliations:** 1 Department of Stomatology, Taihe Hospital and Hubei Key Laboratory of Embryonic Stem Cell Research, School of Basic Medical Sciences, Hubei University of Medicine, Shiyan, Hubei P.R. China; 2 Laboratory of Tumor biology, Academy of Bio-Medicine Research, Hubei University of Medicine, Shiyan, Hubei P.R. China; 3 Department of Clinical Oncology, Taihe Hospital, Hubei University of Medicine, Shiyan, P.R. China

**Keywords:** ELF3-AS1, SNAI2 overexpression, Transcriptional regulation, mRNA stability, Gastric cancer metastasis

## Abstract

**Background:**

The biological function of lncRNA ELF3-AS1 remains largely unknown in cancers. The cause of SNAI2 overexpression in tumor metastasis remains largely unclear. The molecular mechanisms underlying the high co-expression of antisense lncRNAs and adjacent protein-coding genes remains unclear.

**Methods:**

RNA-seq, CHIP and dual-luciferase reporter assay were performed to identify lncRNAs regulated by SNAI2. MicroRNA-seq and RNA-seq studies were conducted to reveal the biological function of ELF3-AS1 in GC. RNA pulldown and CHIRP assays were conducted to identify the protein that interacts with ELF3-AS1.

**Results:**

A total of 123 lncRNAs were identified to be regulated by SNAI2 in GC by RNA sequencing. The ELF3 gene and antisense lncRNA ELF3-AS1 were both transcriptionally repressed by SNAI2 or SNAI1. Down-regulation of ELF3-AS1 and ELF3 predicted poor prognosis in GC. Nuclear localized lncRNA ELF3-AS1 negatively regulated GC cell cycle progression via suppressing G1/S transition and histone synthesis. ELF3-AS1 mainly inhibited GC metastasis by repressing SNAI2 signaling. Additionally, ELF3-AS1 modulated ELF3 mRNA stability by RNA-RNA interaction. The RNA duplexes formed by ELF3 mRNA and lncRNA ELF3-AS1 directly interacted with the double-stranded RNA (dsRNA) binding protein complex ILF2/ILF3 (NF45/NF90). In turn, the ILF2/ILF3 complex dynamically regulated the expression of ELF3-AS1 and ELF3 by affecting the dsRNA stability.

**Conclusions:**

The SNAI2-ELF3-AS1 feedback loop regulates ELF3 expression at transcriptional and post-transcriptional levels and drives gastric cancer metastasis by maintaining SNAI2 overexpression**.** The ILF2/ILF3 complex plays a critical role in regulating dsRNA stability. In addition, our work provides a direct evidence that head-to-head antisense lncRNAs can share promoters with neighboring coding genes, which make their expression subject to similar transcriptional regulation, leading to high co-expression.

**Supplementary Information:**

The online version contains supplementary material available at 10.1186/s13046-022-02541-9.

## Background

Gastric cancer (GC) is the third most common cause of cancer-related deaths [1, 2]. Although treatments for GC have been greatly improved, the survival remains poor due to the inability to diagnose this cancer in early stage [3, 4]. The most common route of GC metastasis is lymph node metastasis, followed by peritoneal dissemination metastasis and liver metastasis [5, 6]. Approximately one-third of GC patients are diagnosed at an advanced stage with metastasis, and 4–14% have metastatic disease to the liver [7, 8]. Since GC metastasis is often characterized by multiple and diffuse distribution, the vast majority of patients have lost the opportunity of surgical treatment by this time [9–11]. Therefore, there is an urgent need to unravel the molecular mechanisms of tumor metastasis.

The transcription repressor SNAI2 (also known as SLUG), which belongs to the SNAIL transcription factor family, is known to be one of the master regulators of epithelial–mesenchymal transition (EMT) [12]. Accumulating evidence have shown that SNAI2 plays a critical role in driving tumor metastasis by transcriptionally regulating downstream target genes [13, 14]. For examples, Olmeda and colleagues have confirmed that Snai1 and Snai2 collaborate on tumor growth and metastasis properties of mouse skin cancer cells [15]. Fan and colleagues have reported that SNAI2 promoted EMT signaling in a non-canonical signaling pathway by transcriptionally repressing miR-222-3p expression ovarian cancer [16]. Transcription factor SNAI2 functioned pro-tumorigenic roles in glioma stem cells via transcriptionally repressing PHLPP2 expression [17]. Thus, uncovering the target genes of SNAI family is essential to better understand tumor metastasis.

Long non-coding RNAs (lncRNA) are a group of classic endogenous non-coding RNAs with a length of more than 200 nucleotides [18]. Recent studies have shown that lncRNA dysregulation plays critical roles in tumorigenesis and metastasis [18–20]. Transcription is the first and most heavily regulated step in gene expression [21]. Like protein-coding genes, the expression of lncRNA and microRNA is also under the regulation of corresponding transcription factors. That means, the abnormal expression of transcription factors usually leads to the aberrant expression of downstream lncRNAs, coding-genes and miRNAs. For example, we previously had confirmed that the down-regulation of the epithelial transcription factor ELF3 leads to the overexpression of the oncogenic lncRNA UBE2CP3 in GC [20].

Antisense lncRNA ELF3-AS1 (also known as SCAT7 or RP11-465N4.4 or ENSG00000234678) is located at chromosome 1q32.1 and broadly expressed in human tissues. ELF3-AS1 was reported to be a cell cycle associated lncRNA that was enriched in S-phage [22]. Recently, lncRNA ELF3-AS1 has been reported to function as an oncogene in lung cancer [23, 24]. However, the biological function of ELF3-AS1 in GC remains unclear. In this study, we comprehensively explored the biological function of lncRNA ELF3-AS1 in GC. Our finding highlights that SNAI2 formed a double-negative feedback loop with lncRNA ELF3-AS1 to maintain self-overexpression, thereby driving GC metastasis.

## Methods

### Cell transfection and establishment of cell lines

Human gastric cancer cell lines AGS, NCL-N87, MGC803 and HGC-27 were purchased from GeneChem (Shanghai, China). The human gastric cancer cell lines (BGC823, SGC7901 and MKN45) and the normal gastric cell line GES-1 were purchased from the Shanghai Cell Bank of the Chinese Academy of Sciences. The siRNAs listed in Table S1 were designed and synthesized by Genepharma (Shanghai, China). Briefly, GC cell lines were seeded into 6-well plates and grown overnight. The next day, when the cell plating density reached 20%-30%, GC cells were transfected with siRNAs (final concentration, 50 nM) by Lipofectamine 2000 (Invitrogen, USA) according to the manufacturer’s instructions. The lentiviruses for knockdown of ELF3-AS1 (C06003) were purchased from Genepharma. The lentiviruses for overexpression of SNAI1 and SNAI2 (GV358, Ubiquitin promotor) were purchased from GeneChem. Transfection was performed according to the manufacturer’s instructions. At the indicated time points, the cells were harvested for mRNA and protein analysis as well as for other assays.

### Clinical GC samples

The study protocol was approved by the Human Research Ethics Committee of Hubei University of Medicine (2018-TH-035). The procedures were in accordance with the Helsinki Declaration of 1975. Written informed consent was obtained from all patients. Tissue samples were immediately frozen in liquid nitrogen after resection and stored at − 80 °C until use. All samples were pathologically confirmed.

### RNA sequencing

The total RNA in GC cells was extracted to perform RNA sequencing (RNA-seq). A total amount of 1.5 μg RNA per sample was used as input material for the RNA sample preparations. The whole step of library construction and sequencing was performed at Shanghai Lifegenes Technology Co., Ltd. The RNA-seq data was uploaded on the GEO section of the NCBI web server. The GEO accession numbers were GSE161551 (SNAI2/SNAI1 overexpression), GSE161291 (ELF3-AS1 knockdown) and GSE161544 (ILF2/ILF3 knockdown).

### MicroRNA sequencing

After knocking down ELF3-AS1 expression in GC cells, the total RNA of each sample was extracted and then sent to BGI company (Wuhan, China) for microRNA purification and miRNA sequencing analysis. The normalized expression level of each microRNA was calculated by TPM value. The Log2FC value was calculated to estimate between-group differences. For each microRNA, if the fold change was more than 1, the difference between the negative control and the matched target samples was set to be significant. The microRNA-seq data was uploaded on the GEO section of NCBI web server. The gene expression omnibus accession number is GSE161553.

### Mouse xenograft model

Four-week-old female BALB/c nude mice were purchased from the Laboratory Animal Center of the Hubei University of Medicine and maintained in a temperature-controlled (21 °C) and light-controlled pathogen-free animal facility with free access to food and water. All animals were treated in accordance with guidelines of the Committee on Animals of the Hubei University of Medicine. GC cells (5 × 10^6^) were injected into the subcutaneous tissue of female BALB/c nude mice. After 28 days, all the mice were ethically euthanized, and the tumors were collected for weighing and volume measurement. The tumor volume was calculated using the following formula: volume = length × (width)^2^/2. The study protocol was approved by the Experimental Animal Research Ethics Committee of Hubei University of Medicine (2019-056).

### RNA Fish assay

Briefly, GC cell lines were seeded and fixed with 4% paraformaldehyde. The next day, when the cell plating density reached 50%-70%, GC cells were treated with 0.5% Triton followed by pre-hybridization. Overnight hybridization was performed with a 10 mM probe concentration. The RNA FISH kit (C10910) was purchased from RiboBio (Guangzhou, China). The experiment was performed according to the manufacturer’s instructions. The 5’FAM-ELF3-AS1 probes were designed and synthesized by Sangon Biotech (Shanghai). The images were taken with a confocal microscope (Zeiss).

### Chromatin immunoprecipitation assay

Chromatin immunoprecipitation (CHIP) assays were performed using a CHIP Assay Kit (56383S , Cell Signal Technology, USA) according to the manufacturer’s protocol. Briefly, the SGC7901 cell line was collected and fixed for 10 min at 37 with 1% formaldehyde, followed in sequence with SDS lysis and DNA shearing, protein and DNA immunoprecipitation, cross-linked DNA reversal and DNA purification. Finally, the immunoprecipitated DNA fragments were detected by PCR assays. The normal rabbit IgG was used as the negative control.

### Western blot assay

Gastric cancer cells were lysed in RIPA buffer with 1 mM PMSF. Total protein (10-100 μg) was electrophoresed through 10% SDS polyacrylamide gels and was then transferred to a PVDF membrane. After blocking with skim milk at 4 °C for 1h, the membrane was incubated with primary antibody at 4°C overnights. The blots were then washed and incubated with horseradish peroxidase (HRP)-conjugated secondary antibody (1: 10000, Earthox) for 1.5 h at room temperature. Detection was performed using a SuperLumia ECL HRP Substrate Kit (Abbkine) and visualized using a Bio-Rad Imaging System (USA). The primary antibodies used in this study were ILF2 (14714-1-AP, 1:5000, Proteintech, Wuhan, China), ILF3 (13099-1-AP, 1:5000, Proteintech), ELF3 (HPA003316, 1:5000, Sigma, USA), β-Actin (66009-1-Ig, 1:10000, Proteintech), TARBP2 (15753-1-Ig, 1:100, Proteintech), SNAI2 (12129-1-AP, 1:1000, Proteintech), SNAI1 (13099-1-AP, 1:500, Proteintech), YBX1 (20339-1-AP, 1:1000, Proteintech).

### RNA Immunoprecipitation assay

After crosslinking with 0.5% formaldehyde for 10 min at room temperature, cells were harvested and lysed in RIP lysis buffer with RNasin (1000 U/ml), DNase I (50 U/ml) and protease inhibitor cocktail. After the genomic DNA was digested, lysates were further subjected to sonication. Supernatants cleared by centrifugation were incubated with the anti-ILF3 antibody (Proteintech, China) or IgG overnight at 4 °C. Protein A/G beads were added for a further 4 h incubation at room temperature. After the beads were washed, immunocomplexes of proteins and RNAs were de-crosslinked at 95 °C for 15 min. The immunoprecipitated RNAs were then purified for RNA sequencing and qRT-PCR analysis.

### RNA pull-down assay

Briefly, 831 bp length of sense and antisense ELF3-AS1 sequences were cloned into pGEM-T Easy (Promega). In vitro transcription was carried out and RNA was purified and labeled with Biotin at 3′ ends. Cells were harvested and resuspended in freshly prepared lysis buffer supplemented with 50 U/mL RNase inhibitor (Takara) and a protease/phosphatase inhibitor cocktail (Roche). Sense and antisense RNAs were captured on magnetic beads (Pierce) and were incubated with cell lysates in protein-RNA-binding buffer (Thermo Scientific) overnight at 4 °C with agitation. RNA-binding protein complexes were washed five times with ice-cold wash buffer and were boiled in SDS lysis buffer for western blot assay and mass spectrometry (MS) analysis.

### Chromatin isolation by RNA purification (CHIRP) assay

Briefly, the CHIRP assay was carried out to verify the interaction between ELF3-AS1 and RNA binding proteins. A 3′ end Biotin modified-DNA probe targeting ELF3-AS1 was designed and synthesized by Sangon. Before crosslinking, SGC7901 cells were grown and seeded into 10 cm dishes. Cell lysates were harvested after incubation of 48 h with a confluence of 60-80%. Cells were cross-linked with 1% formaldehyde and sonicated for the hybridization reaction. After the chromatin was sheared into 100–500 bp fragments, the cell lysates were incubated with the biotinylated DNA probe solution for 4h at 37 °C. The binding complex was covered with streptavidin-conjugated magnet beads. The proteins were finally eluted and purified from the magnet beads for real-time qPCR or mass spectrometry and western blotting analyses.

### Statistical analysis

For gene expression analysis of different subtypes of GC, the P values were estimated using Mann–Whitney nonparametric test. Survival curves were calculated using the Kaplan–Meier method, and differences between the curves were analyzed using the log-rank test. All the rest of the experiments were used an unpaired *t*-test or one-way ANOVA test. For all experiments, a minimum of triplicates per group and repetition of at least three times was applied to achieve reproducibility. All tests with p values less than 0.05 were considered statistically significant.

## Results

### Exploration of lncRNAs regulated by the transcription repressor SNAI2 in GC

**Fig. 1:**
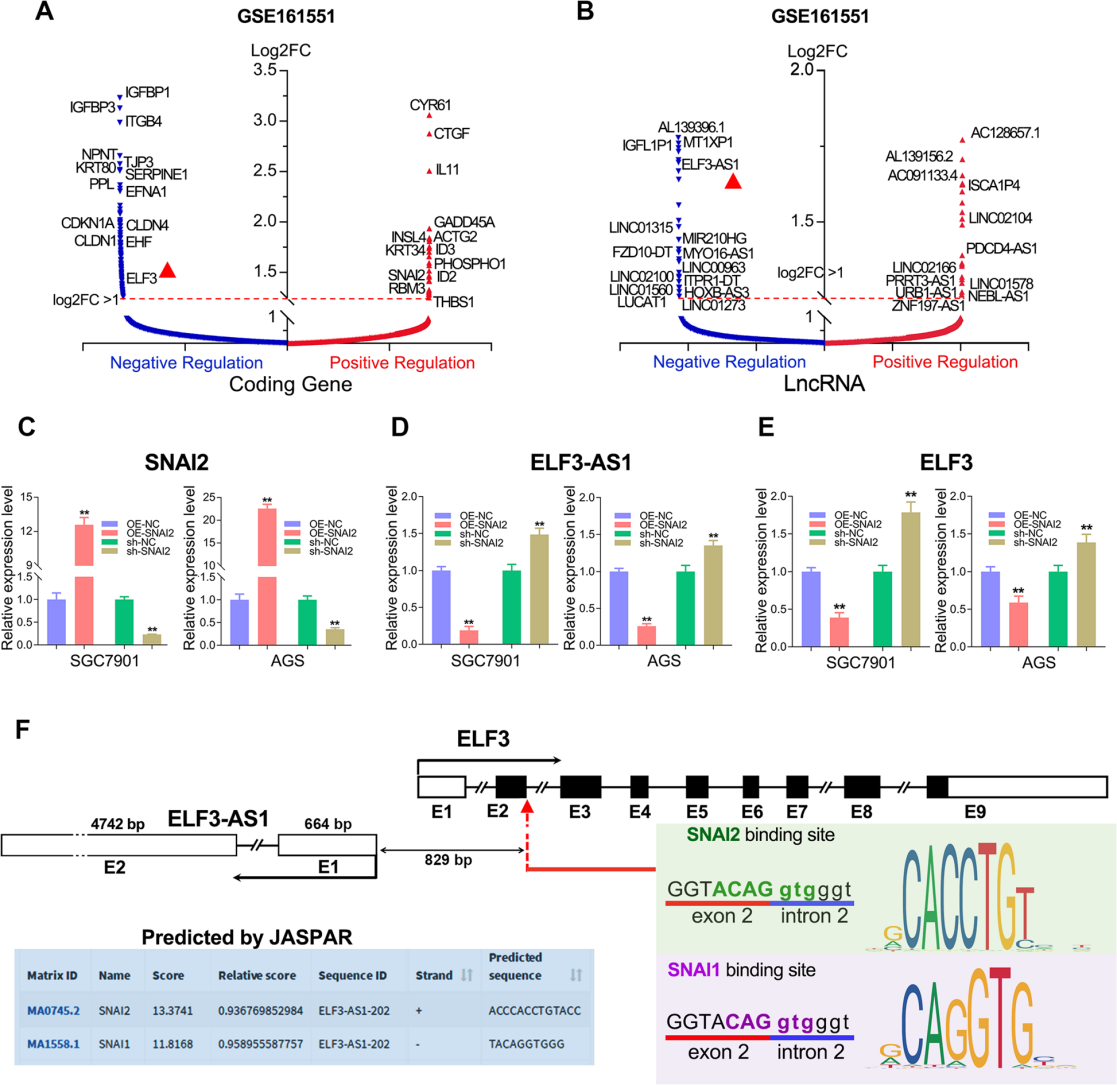
Identification of the SNAI2-regulated lncRNAs in GC by RNA-seq. **A**, **B** The protein-coding genes and the lncRNAs that positively (Red) or negatively (Blue) regulated by SNAI2 were shown in volcano plots (log2FC>1). **C** The overexpression and knockdown efficiency of SNAI2 in the SGC7901 and AGS cell lines were verified by qRT-PCR. **D**, **E** The ELF3-AS1 and ELF3 expression were detected after overexpression/knockdown of SNAI2 in GC cell lines. **F** The promoter analysis by the JASPAR web tool revealed that SNAI2 and SNAI1 could bind on the common promoter of lncRNA ELF3-AS1 and ELF3. **, *P* < 0.01

To explore the target genes of SNAI2, RNA sequencing studies (GSE161551) were conducted in the GC cell line (SGC7901) overexpressing SNAI2. A total of 318 coding genes, 70 lncRNAs were strongly repressed by SNAI2, while 55 coding genes and 53 lncRNAs were greatly upregulated by SNAI2 (|Log2FC| >1, Table S2). As shown in Fig. 1A, the expression of IGFBP1/3, ITGB4, CLDN1/4, TJP3, EFNA1, KRT80, ELF3, etc. were strongly repressed by SNAI2, while the expression of CYR61, CTGF, IL11, ID2/3, RBM3, THBS1 etc. were strongly upregulated by SNAI2. Similarly, around 123 lncRNAs, including linc01315, MIR210HG, FZD10-DT, linc00963, HOXB-AS3, LUCAT1, ITPR1-DT, PDCD4-AS1, ZNF197-AS1, etc., greatly altered their expression after overexpressing SNAI2 (|Log2FC| >1). Interestingly, antisense lncRNA ELF3-AS1 as well as its adjacent gene ELF3 were both strongly repressed by SNAI2 (Fig. 1B).

To further confirm whether ELF3-AS1 and ELF3 could be negatively regulated by SNAI2, loss-of-function and gain-of-function studies regarding SNAI2 were performed in two GC cell lines. As expected, both ELF3-AS1 and ELF3 were significantly downregulated in the SNAI2 overexpression cell lines but were significantly upregulated in the SNAI2-depletion cell lines (Fig. 1C-E). These results suggested that both ELF3-AS1 and ELF3 were negatively regulated by SNAI2 in GC.

ELF3-AS1 is an antisense lncRNA (head-to-head) of the epithelial tumor suppressor gene *ELF3*. Promoter analysis revealed that ELF3-AS1 promoter contained a sequence “GGTACAGGTGGGT”, predicted to be recognized by both SNAI2 and SNAI1. This sequence was located 829 bp upstream of the ELF3-AS1 transcription start point, which was also the junction point between exon 2 and intron 2 of the ELF3 gene (Fig. 1F). Thus, we speculated that both ELF3-AS1 and ELF3 might be transcriptionally regulated by SNAI2 and SNAI1.

### ELF3-AS1 and ELF3 were transcriptionally repressed by both SNAI2 and SNAI1

**Fig. 2:**
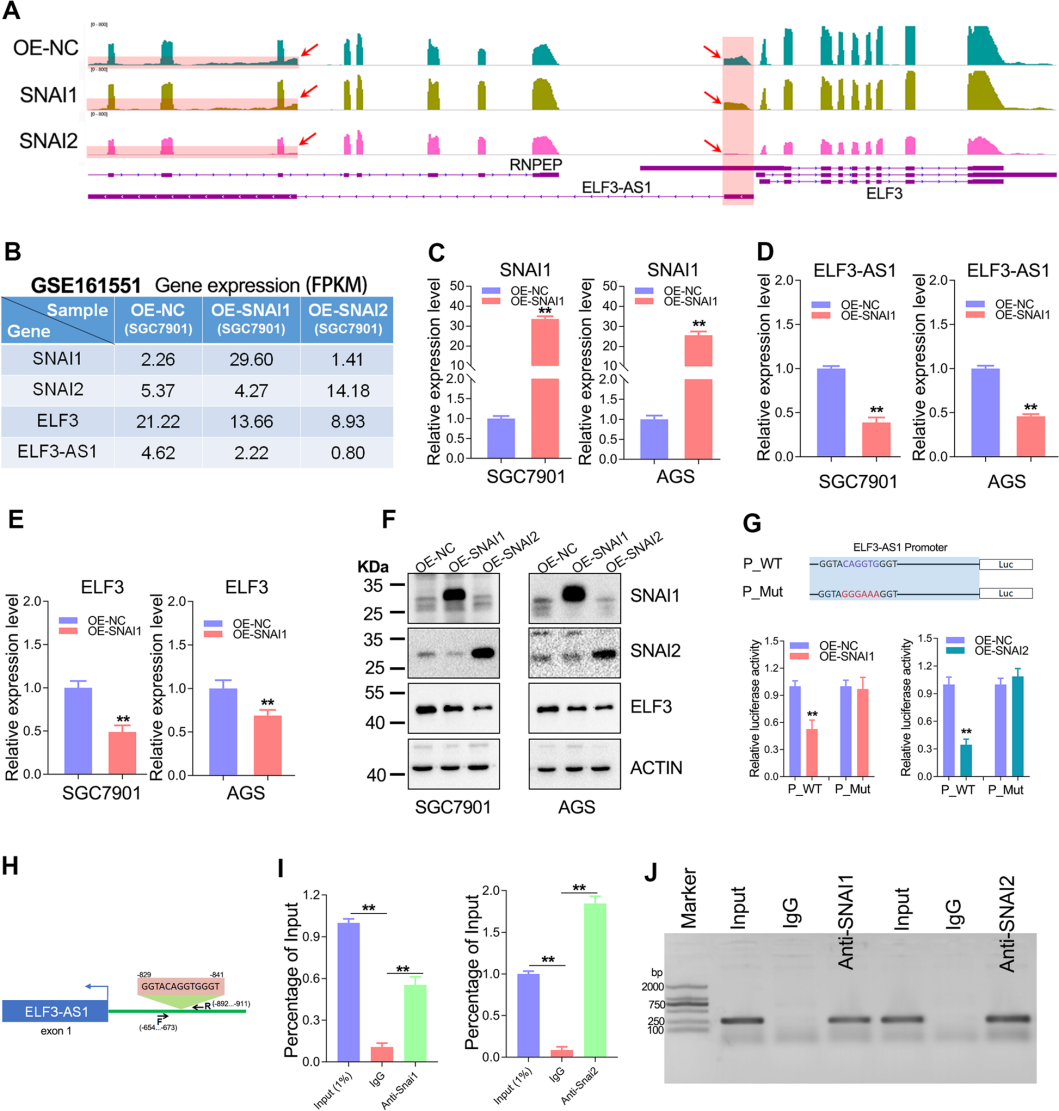
ELF3-AS1 and ELF3 are transcriptionally inhibited by both SNAI2 and SNAI1 in GC. **A** The transcripts abundance of ELF3-AS1 and ELF3 in SNAI2/SNAI1 overexpression cell lines was detected by RNA-seq. **B** The normalized expression (FPKM value) of SNAI1, SNAI2, ELF3 and ELF3-AS1 were shown in the plot. **C** The overexpression efficiency of SNAI1 in GC cell lines was verified by qRT-PCR. **D**, **E** The ELF3-AS1 and ELF3 expression were detected after overexpression of SNAI1. **F** Overexpression of SNAI1 or SNAI2 significantly decreased the protein level of ELF3. **G** Dual-luciferase reporter assay showed that the predictive binding site of SNAI1 or SNAI2 is necessary for their inhibition on Luc expression. **H** Diagram showing the primers location used in CHIP-qPCR/PCR. **I**, **J** Chip assay showed that both SNAI1 and SNAI2 could bind to the promoter of ELF3 or ELF3-AS1. **, *P* < 0.01

LncRNA ELF3-AS1 is abundant in human cells and can be effectively captured by magnetic beads when RNA sequencing. In order to make our results more convincing, we used RNA-seq studies to visualize the expression of ELF3-AS1 and ELF3. As expected, RNA-seq analysis and qRT-PCR assay together showed that ELF3-AS1 and ELF3 were downregulated by SNAI2/SNAI1 overexpression (Fig. 2A-F). Besides, the inhibitory intensity of SNAI2 on the expression of ELF3 and ELF3-AS1 was much greater than that of SNAI1 (Fig. 2B, E).

Furthermore, to figure out whether the repression of ELF3/ELF3-AS1 by SNAI2/SNAI1 occurred at the transcriptional level, dual-luciferase and CHIP assays were performed in the SNAI1 and SNAI2 overexpression cell lines. As shown in Fig. 2G, when the SNAI1/2 binding sequence was mutated, the strong repression of SNAI1/2 overexpression on luciferase expression was partially restored, suggesting that this sequence was necessary for SNAI1/SNAI2 to inhibit ELF3/ELF3-AS1 expression. On the other hand, CHIP assays showed that SNAI1 and SNAI2 could directly bind to the ELF3-AS1 promoter (Fig. 2H-J). Taken together, both SNAI2 and SNAI1 were involved in the transcriptional regulation of ELF3 and ELF3-AS1 in GC.

### The reduced ELF3-AS1 and ELF3 expression predicted poor prognosis in GC

**Fig. 3:**
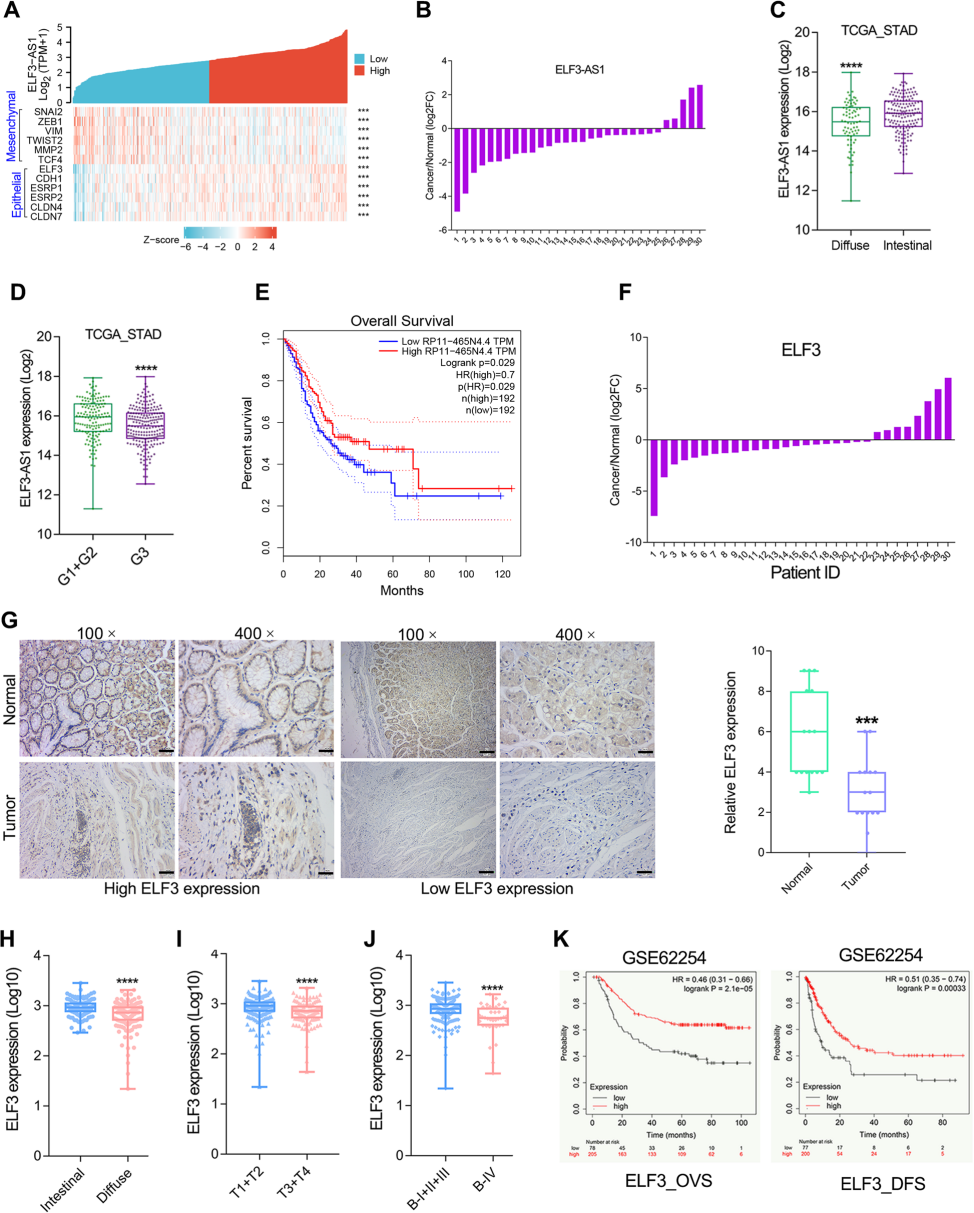
Reduced expression of ELF3-AS1 and ELF3 correlates with poor prognosis in GC. **A** ELF3 and ELF3-AS1 were EMT-related genes that highly co-expressed with epithelial biomarkers in GC. **B** The ELF3-AS1 expression in 30 paired of GC tissues and adjacent non-tumor tissues was examined by qRT-PCR. **C**, **D** The ELF3-AS1 expression in GC patients with different histopathological types and differentiation degrees. **E** Overall Survival analysis using GEPIA web tool showed that ELF3-AS1 predicts favorable prognosis in GC. **F** The ELF3 expression level in 30 paired of GC tissues and adjacent non-tumor tissues was examined by qRT-PCR. **G** Representative IHC images on the tissue microarray (TMA) probed with the anti-ELF3 antibody (scale bars=100 μm or 25 μm, respectively) are shown. **H** The difference in expression levels of ELF3 between intestinal GC tissues and diffuse GC tissues. **I**, **J** ELF3 expression level in different T-stages and Borrmann-stages of GC tissues from the GSE62254 cohort. **K** Kaplan-Meier survival curves of overall survival and disease-free survival based on ELF3 expression in the GC cohort GSE62254. **, *P* < 0.01. ***, *P* < 0.001. ****, *P* < 0.0001

Since ELF3 and ELF3-AS1 were repressed by EMT-related transcription factors SNAI1/2, we further conducted the gene expression correlation analysis between ELF3/ELF3-AS1 and EMT biomarkers. As expected, both ELF3 and ELF3-AS1 were highly co-expressed with epithelial biomarkers (Fig. 3A).

Besides, analysis of ELF3-AS1 expression in 30 pairs of GC tissues showed that ELF3-AS1 was down-regulated in more than 80% of GC samples compared to the corresponding normal samples (Fig. 3B). On the other hand, we analyzed the clinical significance of ELF3-AS1 downregulation in GC samples from the cancer genome atlas (TCGA, *n*=375) database. LncRNA ELF3-AS1 was low expressed in diffuse and poorly differentiated GC tissues (Fig. 3C and D, *p*<0.0001). However, no significant changes in ELF3-AS1 expression levels were observed between GC patients with different TNM stages (data not shown). Overall survival analysis showed that GC patients with a lower expression level of ELF3-AS1 possessed a shorter overall survival time (Fig. 3E, *p*=0.029).

Our previous studies had implied that ELF3 plays tumor-suppressive roles in GC [10, 20]. Herein, we further confirmed that ELF3 expression was significantly downregulated in GC (Fig. 3F, G). Besides, lower expression of ELF3 was observed in the diffuse GC compared with intestinal GC (Fig. 3H). The low expression of ELF3 was positively correlated with the malignant progression of GC (Fig. 3I, J). GC patients with lower ELF3 expression had a poorer overall survival time and disease-free survival time in the GSE62254 GC cohort (Fig. 3K). Conversely, both SNAI2 and SNAI1 were overexpressed, and predicted poor prognosis in GC (Figure S1A-F). The clinical analysis fitted very well with our findings that ELF3-AS1 and ELF3 were transcriptionally repressed by SNAI2 and SNAI1 in GC.

### Epithelial transcription factor ELF3 functioned tumor-suppressive roles in GC

**Fig. 4:**
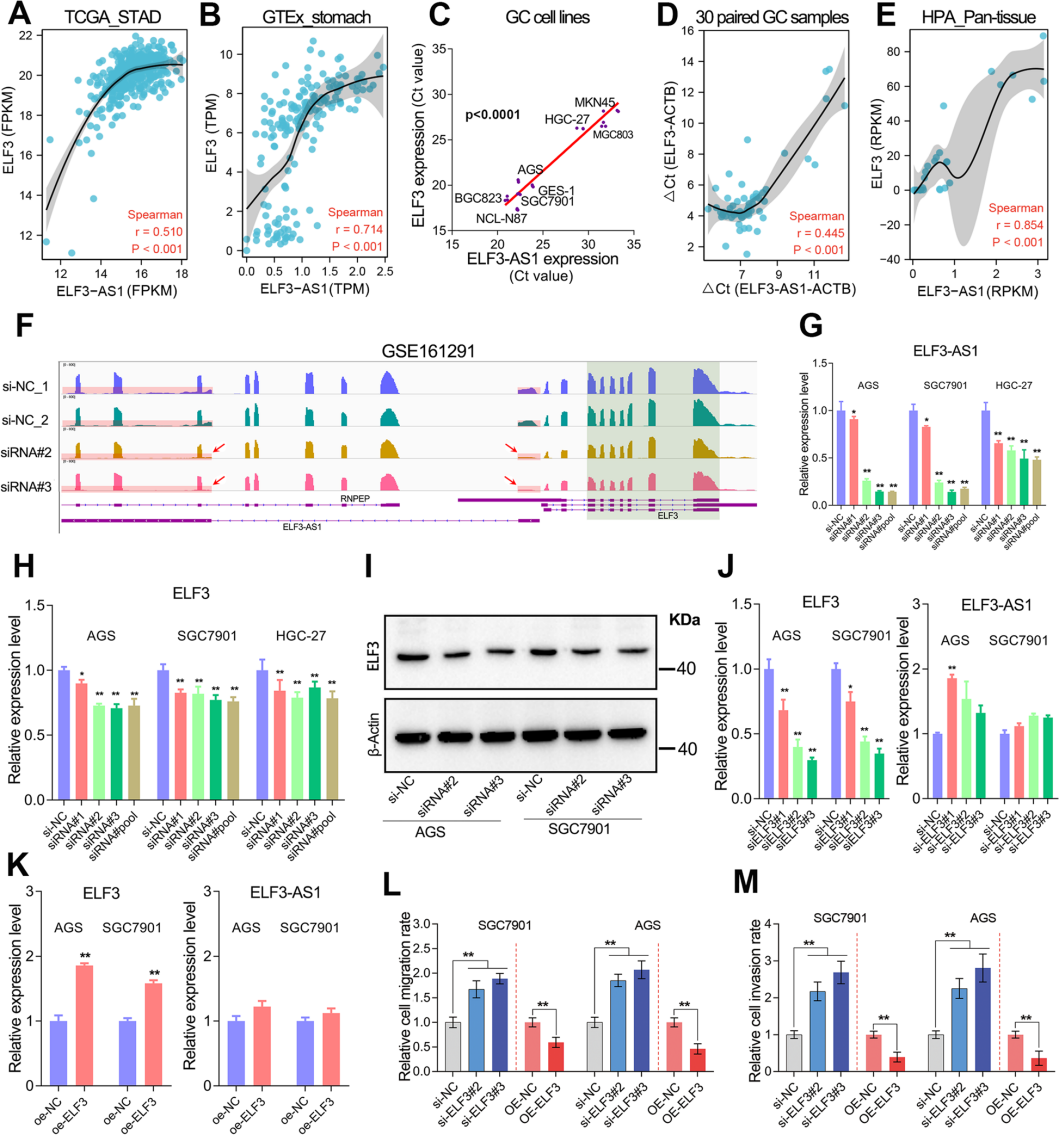
ELF3 negatively regulates cell metastasis but cannot regulate ELF3-AS1 expression in GC. **A**-**D** ELF3-AS1 and ELF3 were highly co-expressed in GC cell lines and tissues. **E** ELF3-AS1 and ELF3 were highly co-expressed in human normal pan-tissues. The expression data of ELF3-AS1 and ELF3 were obtained from the NCBI web tool. **F** The transcripts abundance of ELF3-AS1 and ELF3 was detected by RNA-seq in ELF3-AS1-depleted cell lines. **G** The knockdown efficiency of ELF3-AS1 in GC cell lines was verified. **H**, **I** Knockdown of ELF3-AS1 decreased the mRNA and protein levels of ELF3 in GC. **J**, **K** The ELF3 knockdown/overexpression had no obvious effect on ELF3-AS1 expression. **L**, M ELF3 negatively regulates the migration and invasion of GC cells. **, *P* < 0.01

Antisense lncRNAs are usually highly co-expressed with their neighboring protein-coding genes [25]. Similarly, we also observed there was a high co-expression between ELF3-AS1 and ELF3 in the normal stomach tissues, GC cell lines and pan-tissues (Fig. 4A-E, *p*<0.0001). Interestingly, when the ELF3-AS1 was effectively knocked down in GC cell lines, ELF3 mRNA as well as its protein were significantly reduced to approximately 60-70% (Fig. 4F-I). However, it’s not clear how ELF3-AS1 affects ELF3 expression in GC.

Given ELF3 belongs to the ETS transcription factor family, we initially assumed that the co-expression of ELF3-AS1 and ELF3 may be due to ELF3 regulating the expression of ELF3-AS1. To verify this possibility, loss-of-function and gain-of-function studies regarding on ELF3 were performed in two GC cell lines. However, the expression of ELF3-AS1 had no significant alteration after knockdown or overexpressing of ELF3 in GC (Fig. 4J and K). Although transcription factor ELF3 cannot regulate ELF3-AS1 expression, the scratch wound healing assays and transwell assay confirmed that ELF3 functioned tumor-suppressive roles in GC cell migration and invasion (Fig. 4L and M).

### ELF3-AS1 mainly inhibited GC metastasis through repressing SNAI2 signaling

To determine whether ELF3-AS1 has a tumor-suppressive effect in GC, loss- and gain-of-function studies were performed. Cell apoptosis assay showed that depletion of ELF3-AS1 remarkably accelerated early apoptosis of GC cells (Figure S2A). Besides, cell proliferation, transwell and scratch wound healing assays showed that ELF3-AS1 knockdown promotes GC cell proliferation, migration and invasion (Figure. S2B-F). The overexpression of ELF3-AS1 significantly inhibited the proliferation, migration and invasion abilities of HGC-27 cells (Figure. S2G-I). Moreover, we examined the effect of ELF3-AS1 silencing in a xenograft GC model *in vivo*. The tumor growth of GC cells silencing ELF3-AS1 was significantly increased compared to that of the control GC cells (Figure. S2J and K). These results together suggested that ELF3-AS1 inhibited the proliferation and metastasis of GC cells *in vitro* and *in vivo*.

**Fig. 5:**
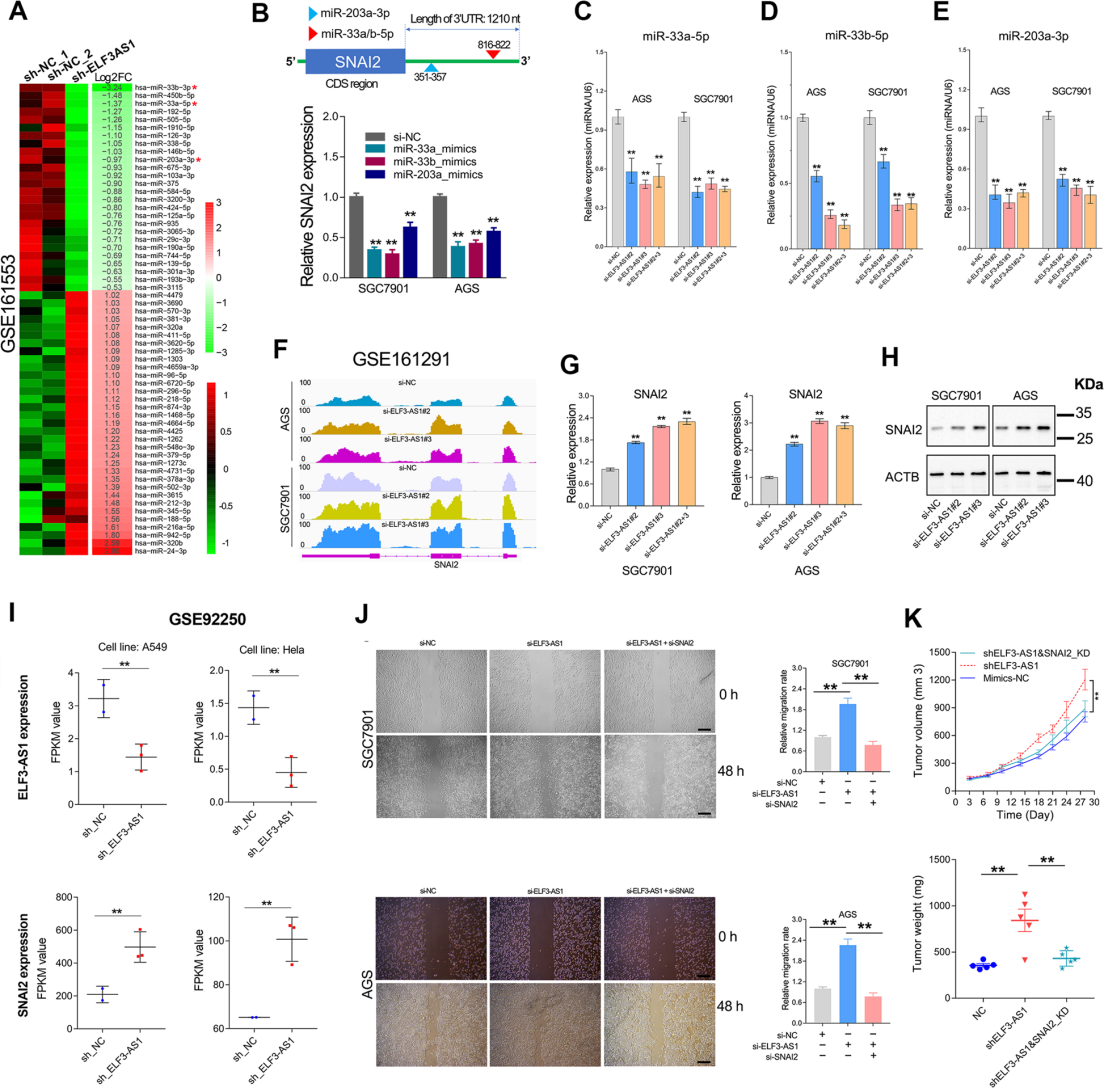
ELF3-AS1 mainly inhibits GC metastasis through repressing SNAI2 signaling. **A** The heat map reveals the differential expressed miRNAs altered by ELF3-AS1 knockdown (left panel), and the log2FC values (ELF3-AS1/NC) were shown in the right panel. The red asterisk indicates the miRNAs targeting SNAI2. **B** The binding sites of miR-33a/b and miR-203a on 3’UTR of SNAI2 were predicted by the TargetScan web tool. SNAI2 mRNA was significantly decreased after overexpression of miR-33a, miR-33b or miR-203a, respectively. **C**-**E** The expression level of miR-33a, miR-33b and miR-203a were detected in the ELF3-AS1 knockdown cell lines. **F** The transcripts abundance of SNAI2 in ELF3-AS1 knockdown cell lines. **G**, **H** The mRNA and protein level of SNAI2 were determined in the ELF3-AS1-depleted GC cell lines. **I** Knockdown of ELF3-AS1 resulted in upregulation of SNAI2 mRNA in A549 and Hela cell lines. The expression levels of SNAI2 and ELF3-AS1 in GSE92250 were normalized to FPKM values. **J**, **K** Rescue assay in vitro and in vivo confirmed that ELF3-AS1 inhibited GC metastasis through SNAI2 signaling. Scale bars=50μm **P < 0.01

Next, we analyzed the differentially expressed miRNAs after ELF3-AS1 knockdown by miRNA sequencing (GSE161553, Fig. 5A). Surprisingly, among the top 10 miRNAs greatly down-regulated by ELF3-AS1 depletion, miR-33a, miR-33b and miR-203a were well-known miRNAs targeting SNAI2 (Fig. 5B). To confirm the reliability of miRNA sequencing, we examined the expression level of miR-33a, miR-33b and miR-203a in the ELF3-AS1 depletion cell lines. The results showed that ELF3-AS1 knockdown can indeed significantly decrease the expression of miR-33a, miR-33b and miR-203a (Fig. 5C-E). Correspondingly, the depletion of ELF3-AS1 led to significant up-regulation of SNAI2 mRNA and protein (Fig. 5F-H). Consistently, analysis of the RNA-seq data from another independent study (GSE92250) also showed that ELF3-AS1 knockdown led to SNAI2 mRNA upregulation in the A549 and Hela cell lines (Fig. 5I), suggesting the negative regulation of SNAI2 expression by ELF3-AS1 might be widely in cancers [22].

In addition, the gene expression profiles of SNAI2 overexpression and ELF3-AS1 knockdown were very similar (Figure S3A). The genes strongly regulated by SNAI2 overexpression also showed similar expression changes in ELF3-AS1-depleted GC cells (Figure S3B-D). For examples, the genes strongly inhibited by SNAI2 overexpression, such as IGFBP3, ITGB4, TJP3, PPL and DDIT4, were also significantly down-regulated in the ELF3-AS1-depleted GC cell lines. The genes strongly induced by SNAI2 overexpression, such as IL11, THBS1, INSL4 and linc02104, were also remarkably upregulated in the ELF3-AS1-depleted GC cell lines. These results strongly implied that knockdown of ELF3-AS1 could not only upregulate SNAI2 expression but also greatly activate the downstream signaling of SNAI2 in GC.

Based on the above findings, we speculated ELF3-AS1 may inhibit GC progression through repressing SNAI2 signaling. To verify this possibility, the rescue assays were performed *in vivo* and *in vitro*. Knockdown of SNAI2 expression rescued the tumorigenic properties of ELF3-AS1-depleted GC cell lines (Fig. 5J and K). These data strongly indicated that ELF3-AS1 mainly inhibited the migration and invasion of GC cells through repressing SNAI2 signaling.

### The nuclear-localized lncRNA ELF3-AS1 plays critical roles in cell cycle progression.

**Fig. 6:**
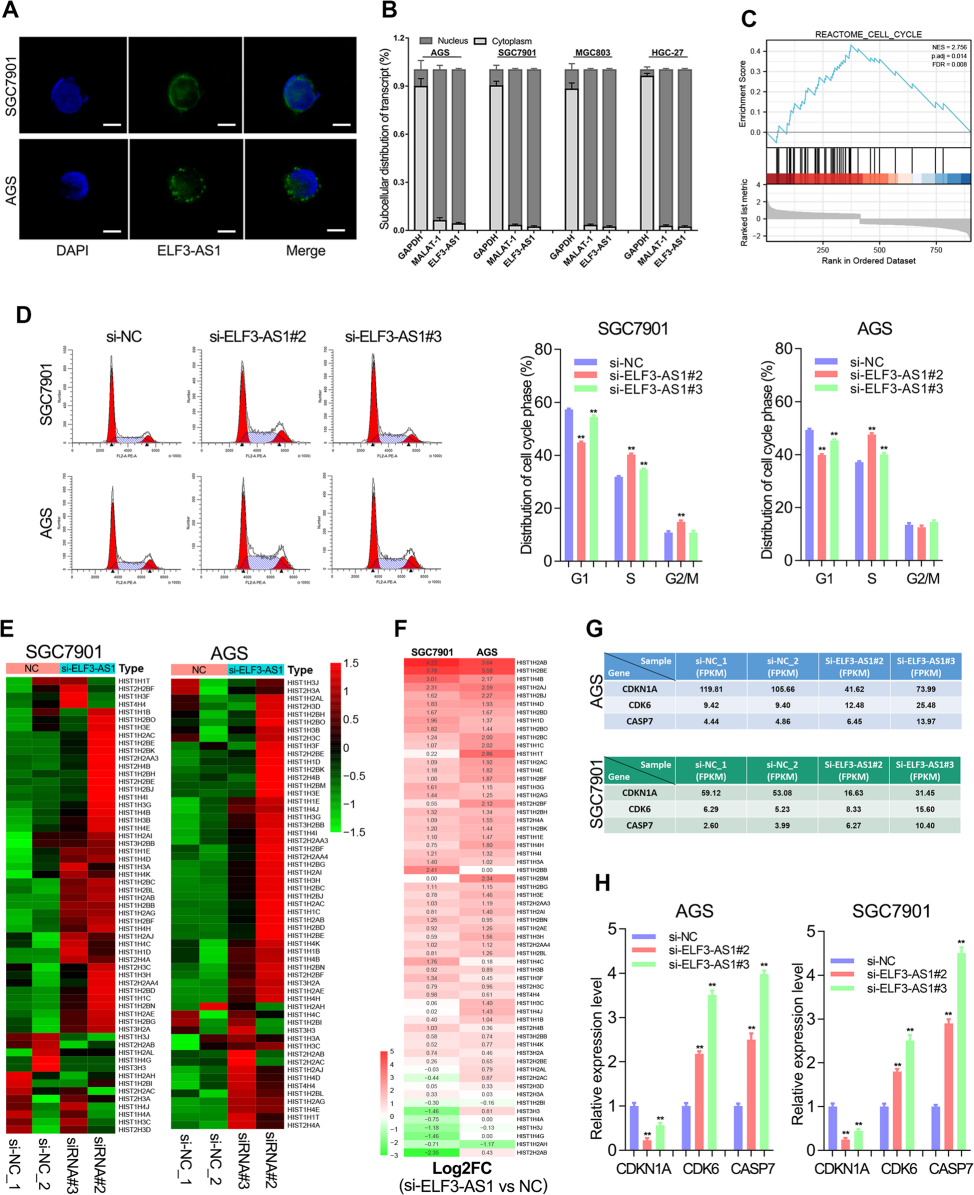
Nuclear-localized lncRNA ELF3-AS1 regulates cell cycle progression by affecting G1/S transition and histone synthesis. **A** Subcellular localization of ELF3-AS1 by RNA FISH in GC cell lines. Bar, 10 μm. **B** The ELF3-AS1 transcripts were mainly located in the nucleus of GC cell lines. **C** GSEA analysis showed that ELF3-AS1 depletion possessed an obvious effect on cell cycle progression in GC (**D**) ELF3-AS1 negatively regulated the G1/S transition of the cell cycle in GC. **E**, **F** The histone-coding genes significantly regulated by ELF3-AS1 knockdown (|Log2FC|>0.7) were shown in the heat map. Almost all the histone-coding genes were upregulated in the ELF3-AS1-depleted cell lines. **G** The normalized expression (FPKM value) of CDKN1A, CDK6 and CASP7 after knockdown of ELF3-AS1 in GC cell lines. **H** The mRNA level of CDKN1A, CDK6 and CASP7 were determined after knockdown of ELF3-AS1. **P < 0.01

The biological function of lncRNAs is closely related to their subcellular location [26]. ELF3-AS1 was a nuclear-localized lncRNA in GC (Fig. 6A and B). A previous study had reported that ELF3-AS1 was a cell cycle-related lncRNA [22]. Our study also showed that lncRNA ELF3-AS1 played essential roles in cell cycle progression. ELF3-AS1 knockdown significantly accelerated the G1/S transition of the cell cycle in GC (Fig. 6C and D). Interestingly, RNA-seq analysis showed that knockdown of ELF3-AS1 resulted in a significant up-regulation of almost all histone-coding genes by more than 2 times (Fig. 6E and F). It’s well known that the synthesis of histones occurs at S phase of the cell cycle, which is synchronized with DNA replication. These results indicated that ELF3-AS1 negatively regulates cell cycle progression of GC cells by affecting G1/S transition and histone synthesis.

To better understand the molecular mechanism of ELF3-AS1 in regulating the cell cycle process, we analyzed the expression changes of cell cycle-related genes after knockdown of ELF3-AS1. The results showed that knockdown of ELF3-AS1 increased the expression of CDK6 and CASP7, but decreased the expression of CDKN1A (also known as p21) in GC (Fig. 6G and H). The p21 proteins functioned as a cell cycle checkpoint of G1/S transition [27]. The CDK6/CCND1 protein complex is very important for cell cycle G1 phase progression and G1/S transition [28]. Therefore, the promotion of G1/S transition caused by ELF3-AS1 knockdown may be due to the downregulation of P21 and the upregulation of CDK6.

### ILF2/ILF3 complex could directly modulate the stability of the ELF3-AS1/ELF3 RNA duplex

**Fig. 7:**
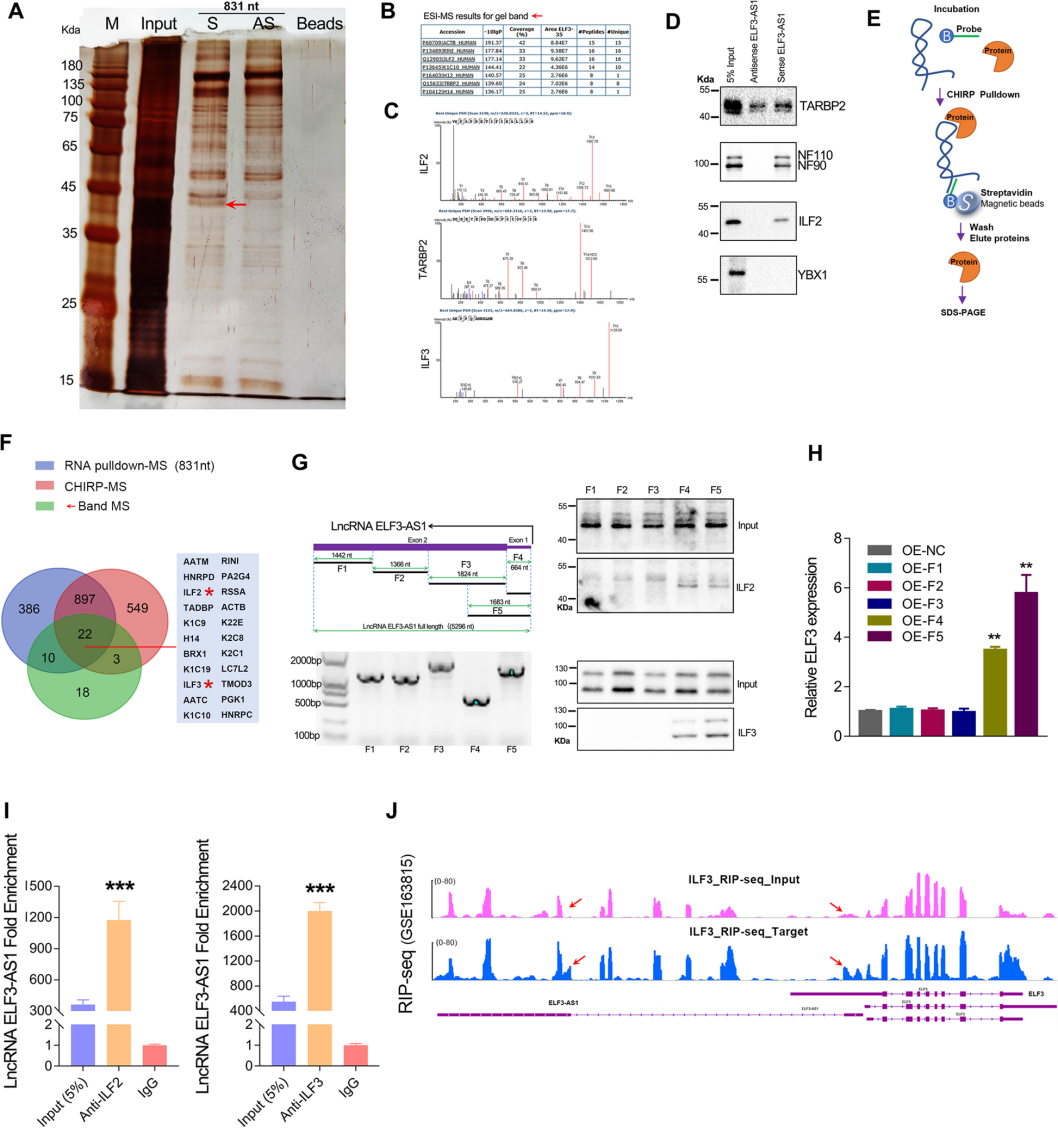
ELF3-AS1 directly interacts with the ILF2/ILF3 protein complex. **A** RNA pull-down assay for identification of ELF3-AS1 binding proteins was conducted. The sense (S) and anti-sense (AS) of ELF3-AS1 RNA were biotinylated, refolded, and incubated with SGC7901 cell lysates. **B**, **C** Identification of ELF3-AS1 binding proteins using a combined RNA pulldown-MS assay. According to the MS analysis of band labeled with a red arrow, RINI, TARBP2, ILF2 and ILF3 were the most possible RNA-binding proteins that interacted with ELF3-AS1. **D** Validation of ELF3-AS1 binding proteins obtained by RNA pulldown using western blotting. **E** Schematic workflow of the CHIRP assay for identification of ELF3-AS1 binding proteins. **F** The Venn plot of the ELF3-AS1 binding proteins identified by three mass spectrometry. **G** Verification of ILF2 and ILF3 protein with western blotting in the precipitation pulled down by different length of ELF3-AS1 RNA fragments. **H** ELF3 expression level in GC cells transfected with different lengths of ELF3-AS1 RNA fragments. **I** RIP-qPCR assay verified the interaction between ELF3-AS1 and ILF2/ILF3. Fold Enrichment was determined relative to IgG control. **J** RIP-seq_anti-ILF3 (GSE163815) assay verified the interaction between ILF3 protein and ELF3/ELF3-AS1 mRNA. **, *P* < 0.01

The potential proteins interacted with lncRNA ELF3-AS1 were identified by RNA pull-down analysis. According to the mass spectrometry (MS) analysis of the differential bands located at 45 Daltons (Fig. 7A), there were 7 proteins with matching coverage greater than 20% (Table S3). Among those proteins, RINI, ILF2 (also known as NF45) and TARBP2, were double-strand RNA (dsRNA) binding proteins (Fig. 7B). Interestingly, another protein named ILF3 (also known as NF90/NF110) was also appeared in the MS results of this band (Fig. 7C). Our subsequent western blot assay further verified that ILF2, ILF3 and TARBP2 could bind to exogenous lncRNA ELF3-AS1 (Fig. 7D). Moreover, CHIRP pulldown assay verified that endogenous ELF3-AS1 was also bound to the ILF2 and ILF3 proteins (Fig. 7E and F). The Venn plot showed that approximately 22 proteins are displayed at the intersection of the three MS results, including ILF2, ILF3, RINI, etc. (Table S3-5, Fig. 7F). To figure out which region of ELF3-AS1 transcript could interact with ILF2 and ILF3, we truncated ELF3-AS1 transcripts of different lengths. After analyzing the proteins pulled down by ELF3-AS1 transcripts of different lengths by western blotting, we found that the first exon region was necessary for the interaction between the ELF3-AS1 transcript and the ILF2/ILF3 complex (Fig 7G). In addition, we detected ELF3 expression in GC cells transfected with ELF3-AS1 transcripts of different lengths. The results showed that only overexpression of ELF3-AS1 transcripts containing exon 1 upregulated ELF3 expression (Fig. 7H). RNA immunoprecipitation assay showed that the ELF3-AS1 transcripts bound to ILF2 and ILF3 proteins were thousands of times higher than the control IgG group (Fig. 7I, J).

**Fig. 8:**
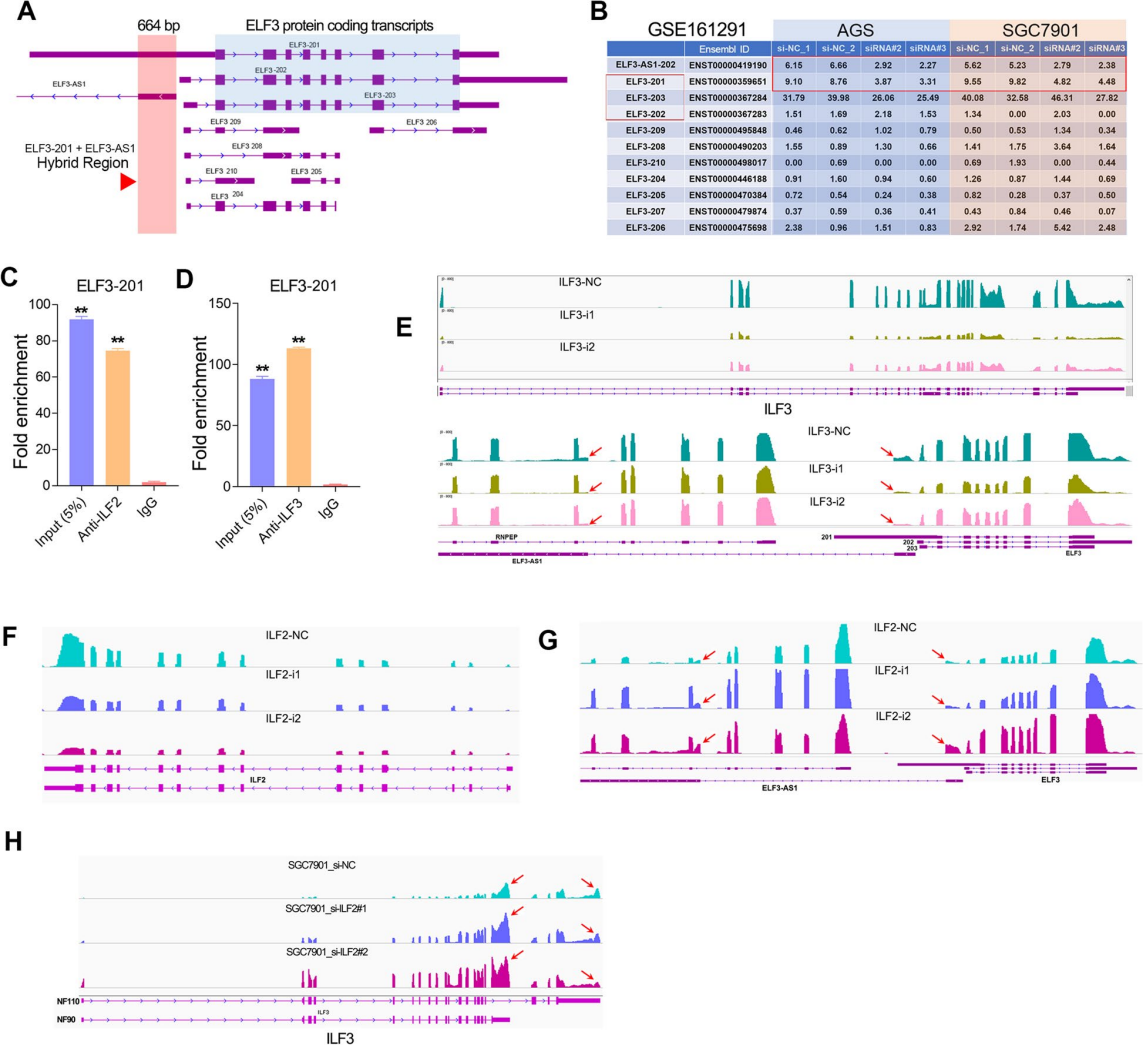
The ILF2/ILF3 interacts with ELF3-AS1/ELF3 RNA duplex to affect RNA duplex stability. **A** The gene structure and location of ELF3-AS1 and ELF3 genes. **B** The normalized expression (FPKM value) of different ELF3 transcripts after knockdown of ELF3-AS1 was shown in the plot. Knockdown of ELF3-AS1 remarkably decreased the expression of ELF3-201 transcripts in GC cell lines. **C**, **D** RIP-qPCR assay verified the interaction between ELF3-201 transcripts and ILF2/ILF3 complex. **E** The transcripts abundance of ELF3 and ELF3-AS1 in ILF3-silenced samples was detected by RNA-seq. (F, G) The transcripts abundance of ELF3 and ELF3-AS1 in ILF2-silenced samples was detected by RNA-seq. (H) ILF2 knockdown affected the alternative splicing of the ILF3 gene. **, *P* < 0.01

The *ELF3* gene has different transcripts due to alternative splicing. Among those different types of ELF3 transcripts, ELF3-201, ELF3-202 and ELF3-203 can encode full-length ELF3 protein. The ELF3-201 transcript and the ELF3-AS1 transcript overlapped by about 664 nucleotides (Fig. 8A). In other words, ELF3-AS1 can combine with the first exon region of ELF3-201 to form a double-stranded RNA molecule. On the other hand, RNA-seq analysis revealed that knockdown of ELF3-AS1 had a more profound effect on ELF3-201 expression compared to the ELF3-203 or any other ELF3 transcripts level (Fig. 8B). These data implied that the ILF2/ILF3 complex may bind to the dsRNA formed by ELF3-AS1 and ELF3-201. To further verify this probability, we also examined the ELF3-201 transcript level in the RIP assays of ILF2/ILF3. The ELF3-201 transcripts bound to ILF2 and ILF3 were much higher than those in the control IgG group (Fig. 8C, D), suggesting that ILF2/ILF3 complex could bind to the dsRNA formed by ELF3-AS1 and ELF3-201.

It has been reported that ILF2/ILF3 complex play an important role in regulating mRNA stability [29]. The RNA-seq data and the qPCR assays indicated that knockdown of ILF3 significantly decreased the mRNA level of ELF3-AS1 and ELF3-201, while knockdown of ILF2 significantly increased the mRNA level of ELF3-AS1 and ELF3-201 (Fig. 8E-G, Figure S4A-F). These results suggested that ILF2 and ILF3 protein possessed opposite effects on the stability of ELF3-AS1 transcripts. A previous study reported that NF45 functions as a regulatory subunit in ILF2/ILF3 complexes [30]. Interestingly, we also noted that knockdown of ILF2 could affect the alternative splicing of ILF3 gene (Fig. 8H). Knockdown of ILF2 significantly upregulate the expression of NF90, but significantly decreased the expression of NF110 (Figure S4G-I). Therefore, we speculated that ILF2 might regulate ELF3-AS1 and ELF3-201 expression through affecting the alternative splicing of the ILF3 gene.

## Discussion

EMT (Epithelial-Mesenchymal Transition) is a key factor leading to the poor prognosis in GC patients. Oh et al. previously reported that GC patients with mesenchymal phenotype has a poorer prognoses compared to the GC patients with an epithelial phenotype [31]. Similarly, Cristescu et al. has reported that GC patients belonging to EMT subtypes possessed the worst prognosis [32]. Our previous studies have identified 3 EMT-related lncRNAs in GC, including UBE2CP3, MAGI2-AS3 and NR2F1-AS1 [18–20]. These work strongly implied that the EMT-related lncRNAs can serve as prognostic biomarkers in GC and play critical roles in gastric carcinogenesis. Thus, uncovering the biological functions of these EMT-related lncRNAs is helpful to better understand the role of abnormal EMT signaling in GC metastasis.

In this study, we took SNAI2 as a breakthrough point to screen lncRNAs regulated by SNAI2 in GC. Overexpression of SNAI2 caused a 2-fold change in the expression of about 123 lncRNAs. ELF3-AS1 was one of the top 10 lncRNAs most significantly repressed by SNAI2 overexpression. Furthermore, ELF3-AS1 and ELF3 gene were identified to be directly repressed by SNAI2 and SNAI1 at the transcriptional level. Although SNAI1 had a similar effect to SNAI2 in inhibiting the expression of ELF3 and ELF3-AS1 or any other downstream target genes, the regulatory efficiency of SNAI1 on the expression of target genes is much weaker than that of SNAI2 (Fig S3). This result indicates that SNAI1 and SNAI2 were functionally redundant in GC and SNAI2 may be the major effect gene in the SNAIL family.

**Fig. 9:**
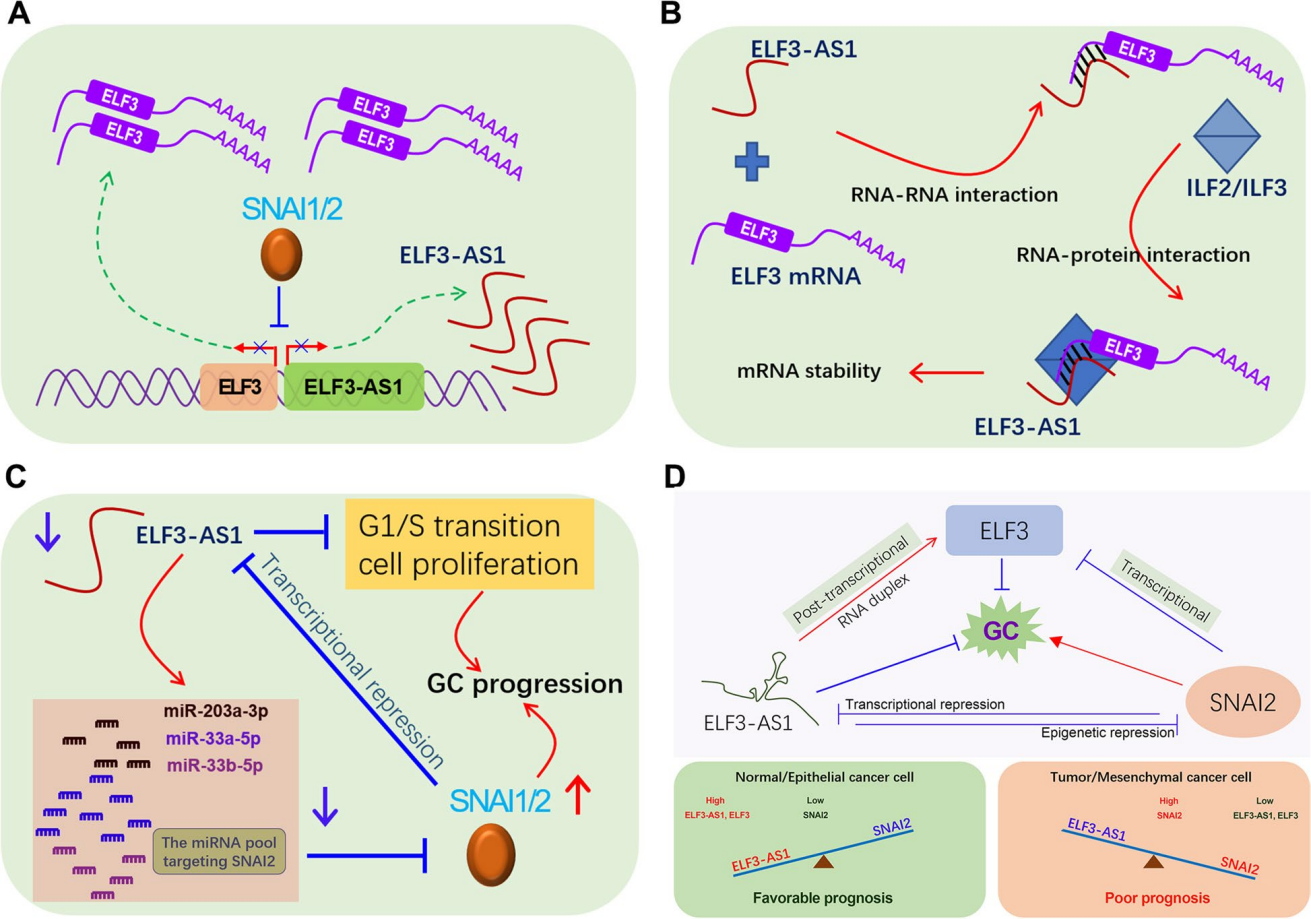
The working model of the SNAI2-ELF3-AS1 feedback loop. **A** ELF3-AS1 and ELF3 shared promoters and were transcriptionally repressed by SNAI1/2. **B** The molecular mechanisms underlying how ELF3-AS1 regulates ELF3 mRNA stability. **C** The molecular mechanisms of the biological role of ELF3-AS1 in GC. **D** The working model of SNAI2-ELF3-AS1 feedback loop in driving GC progression

The ways led to the co-expression of antisense lncRNAs and nearby protein-coding genes are diverse, including transcriptional level and post-transcriptional level. At the transcriptional level, a possible mechanism is that antisense lncRNAs share a promoter with nearby genes, thereby suffering from similar transcriptional regulation. Bartl and colleagues have reported that lncRNA HHIP-AS1 and HHIP shared promoters, and this promoter activity was negatively regulated by DNA methylation [33]. Herein, we also found that ELF3-AS1 and ELF3 genes shared the same promoter, which makes them both under the repression of SNAI2 and SNAI1 (Fig 9A). At the post-transcriptional level, antisense lncRNA can regulate adjacent gene expression by RNA-RNA interactions to avoid mRNA degradation [34]. Herein, we found lncRNA ELF3-AS1 directly stabilized ELF3 mRNA by forming a dsRNA with their overlapping region. Besides, the ILF2/ILF3 complex could dynamically regulate the dsRNA stability of ELF3-AS1 and ELF3 (Fig 9B), suggesting ILF2/ILF3 complex may play a critical role in modulating dsRNA stability. Interestingly, a recent publication by Mahale and colleagues provided very similar evidence for the role of antisense RNA (IER3-AS1) and sense RNA (IER3 )[35]. Based on RNA-seq analysis, they found that activation of FGF2/FGFR signaling greatly enhanced the mRNA levels of IER3 and IER3-AS1. IER3 and IER3-AS1 formed RNA duplex and regulated their mRNA stability each other by interacting with HNRNPK protein [35].

The biological effects of transcriptional repression of ELF3-AS1 by SNAI2 remains unknown. SNAI2 is a rapid-turnover protein [36]. Recently, Kang et al. had reported that SNAI2 protein turnover was regulated by the ubiquitin-proteasome system (UPS) [12, 13]. However, in theory, the regulation of SNAI2 protein turnover should be not only at the (post-) translational level, but also at the (post-) transcriptional level. Herein, our finding strongly indicated that the SNAI2-repressed lncRNA ELF3-AS1 played an essential role in maintaining SNAI2 mRNA stability. Knockdown of ELF3-AS1 results in decreased expression levels of miRNAs targeting SNAI2, upregulation of SNAI2 mRNA and protein, and activation of downstream signaling of SNAI2. Additionally, the overall survival analysis based on the TCGA data showed that ELF3-AS1 and SNAI2 possessed opposite prognoses in pan-cancer (Figure S5). These findings highlighted that SNAI2 achieves self-overexpression by transcriptionally repressing ELF3-AS1. Once SNAI2 is overexpressed, it can transcriptionally repress ELF3-AS1 expression, thereby maintaining self-overexpression state in tumor metastasis. On the other hand, the downregulation of lncRNA ELF3-AS1 promoted GC cell proliferation by accelerating the G1/S transition and increasing histone-coding gene expression (Fig 9C).

## Conclusions

In summary, a novel double-negative feedback loop between SNAI2 and lncRNA ELF3-AS1 was identified in GC. The SNAI2-ELF3-AS1 feedback loop drives GC metastasis by continuously activating SNAI2 signaling and regulating ELF3 expression at transcriptional and post-transcriptional levels (Fig 9D). In GC, SNAI2 was overexpressed, resulting in decreased expression level of ELF3 and ELF3-AS1. In turn, ELF3-AS1 downregulation further drives tumor progression by continuously activating SNAI2 signaling and promoting cell proliferation, thereby leading to a poor prognosis in GC (Fig 9D).

## Supplementary Information

**Additional file 1.**

**Additional file 2.**

**Additional file 3.**

**Additional file 4.**

**Additional file 5.**

**Additional file 6.**

## Data Availability

The datasets supporting the conclusions of this article are included within the article and its additional files.

## Abbreviations

GC: Gastric cancer
TCGA: The Cancer Genome Atlas
UTR: Untranslated region
EMT: epithelial-mesenchymal transition
TPM: transcripts per million
qRT-PCR: quantitative reverse transcription Polymerase Chain Reaction
GEO: Gene Expression Omnibus
FPKM: Fragments Per Kilobase of exon model per Million mapped fragments
STAD: Stomach adenocarcinoma
HNSC: Head and Neck squamous cell carcinoma
KIRC: Kidney renal clear cell carcinoma
ESCA: Esophageal carcinoma
READ: Rectum adenocarcinoma
LIHC: Liver hepatocellular carcinoma
LUSC: Lung squamous cell carcinoma
CHIRP: Chromatin Isolation by RNA Purification
dsRNA: Double-stranded RNA
