# Supplementary material for: LncRNA ELF3-AS1 inhibits gastric cancer by forming a negative feedback loop with SNAI2 and regulates ELF3 mRNA stability via interacting with ILF2/ILF3 complex
Source: J Exp Clin Cancer Res. 2022 Dec 2;41:332. doi: 10.1186/s13046-022-02541-9 (PMC9716751; doi:10.1186/s13046-022-02541-9)
Supplement: Supplementary file 1 — Additional file 1. [file 13046_2022_2541_MOESM1_ESM.docx]

**The SNAI2-ELF3-AS1 feedback loop drives gastric cancer metastasis and regulates ELF3** **expression at transcriptional and post-transcriptional levels**

**Supplementary Tables**

**Supplementary Table S1:** The information of siRNAs and primers used in this study.

| Gene Name | Sequence (5→`3`) | |
| --- | --- | --- |
| siRNA | | |
| siELF3-AS1#1 | GGCUGACCUGAGUCAGAAATT | UUUCUGACUCAGGUCAGCCTT |
| siELF3-AS1#2 | GGAGAGGAGUUACUAGGUUTT | AACCUAGUAACUCCUCUCCTT |
| siELF3-AS1#3 | GCCAGAGAAUUGGCUACAATT | UUGUAGCCAAUUCUCUGGCTT |
| siELF3#1 | GCUGCAACCUGUGAGAUUATT | UAAUCUCACAGGUUGCAGCTT |
| siELF3#2 | CCUCUGCAAUUGUGCCCUUTT | AAGGGCACAAUUGCAGAGGTT |
| siELF3#3 | CCAUGAGGUACUACUACAATT | UUGUAGUAGUACCUCAUGGTT |
| si-ILF2#1 | CUUUGUACCACAUAUCCCATT | UGGGAUAUGUGGUACAAAGTT |
| si-ILF2#2 | GAACUCCAUUUGGAUAUCATT | UGAUAUCCAAAUGGAGUUCTT |
| si-ILF3#1 | GACCGAAAUUUGCUGCUAATT | UUAGCAGCAAAUUUCGGUCTT |
| si-ILF3#2 | GGAGGUUGAUGGCAAUUCATT | UGAAUUGCCAUCAACCUCCTT |
| qRT-PCR | Forward primer | Reverse primer |
| SNAI2 | GCATTTGCAGACAGGTCAAA | TCCTCATGTTTGTGCAGGAG |
| SNAI1 | AGCGAGCTGCAGGACTCTAA | GGACAGAGTCCCAGATGAGC |
| ILF3-Total | CCCCAGAGGACGACAGTAAA | CTCCTTACACAGCAGCACCA |
| NF110 | CCTTGTCTCACCACCAACCT | CCAGAAGCTCCCAACTATGC |
| NF90 | CGGAGTCATTCTGGCTCTCT | CGCAAAATCTTGCAAGTCAA |
| ILF2 | AACAGTGCCACCCAATCTTC | CCAGGAAAACGAATCCTCAA |
| ELF3 | GAAGTGACGTGGACCTGGAT | CTTCTTGCCCTCGAGACAGT |
| ACTIN | ATCGTCCACCGCAAATGCTTCTA | AGCCATGCCAATCTCATCTTGTT |
| P21 | ttagcagcggaacaaggagt | gccgagagaaaacagtccag |
| P53 | ATGGAGGAGCCGCAGTCAGATC | CCATTGTTCAATATCGTCCGGG |
| CDK6 | AACACCCTTGGTGGCTTATG | TTTCCTTGGAGAAGCAGAGC |
| CASP7 | CACCTATCCTGCCCTCACAT | TTATGGGCCAGGCTTACATC |
| MALAT1 | AAAGCAAGGTCTCCCCACAAG | GGTCTGTGCTAGATCAAAAGGCA |
| GAPDH | TCACCAGGGCTGCTTTTA | AAGGTCATCCCTGAGCTGAA |
| ELF3-201 | ATTGTGTTTCGGGCTGAGTC | CCAGGTATGCAGGTGTGTTG |
| ELF3-AS1 | CGGCTCTGCTTGAAAGTTCT | CTGACTGAACCCAAGCCATT |
| CHIP-primer | Forward primer | Reverse primer |
| ELF3-AS1_P | tcccccatttgtctaacagg | CCTACCCACAGGTAGCCTCA |
| miRNA qPCR | Forward primer | Reverse primer |
| U6 | CTCGCTTCGGCAGCACA | AACGCTTCACGAATTTGCGT |
| miR-33a | gggGTGCATTGTAGTTGCA | CAGTGCGTGTCGTGGAGT |
| miR-33b | gggGTGCATTGCTGTTGCA | CAGTGCGTGTCGTGGAGT |
| miR-203a | gggGTGAAATGTTTAGGACC | CAGTGCGTGTCGTGGAGT |

**Supplementary Table S2:** The differentially expressed protein-coding genes and lncRNAs after overexpressed of SNAI2 and SNAI1 in SGC7901 cells.

**Supplementary Table S3:** The mass spectrometry analysis of the proteins in the differential band pulled down by sense and antisense ELF3-AS1.

**Supplementary Table S4**: The mass spectrometry analysis of the total proteins extracted by RNA pulldown assay.

**Supplementary Table S5:** The mass spectrometry analysis of the total proteins extracted by CHIRP assay.

**Supplementary Figures**

**Supplementary Figure S1** Overexpression of SNAI2 and SNAI1 correlates with poor prognosis in GC. (A, B) The expression of SNAI2 and SNAI1 were upregulated in GC according to the pan-cancer analysis of the available TCGA dataset. (C, D) Kaplan–Meier analysis of overall survival time, disease-free time of GC patients from GSE62254 cohort according to the expression of SNAI2 and SNAI1. (E, F): Kaplan–Meier analysis of overall survival time, disease-free time of GC patients from TCGA cohort according to the expression of SNAI2 and SNAI1.


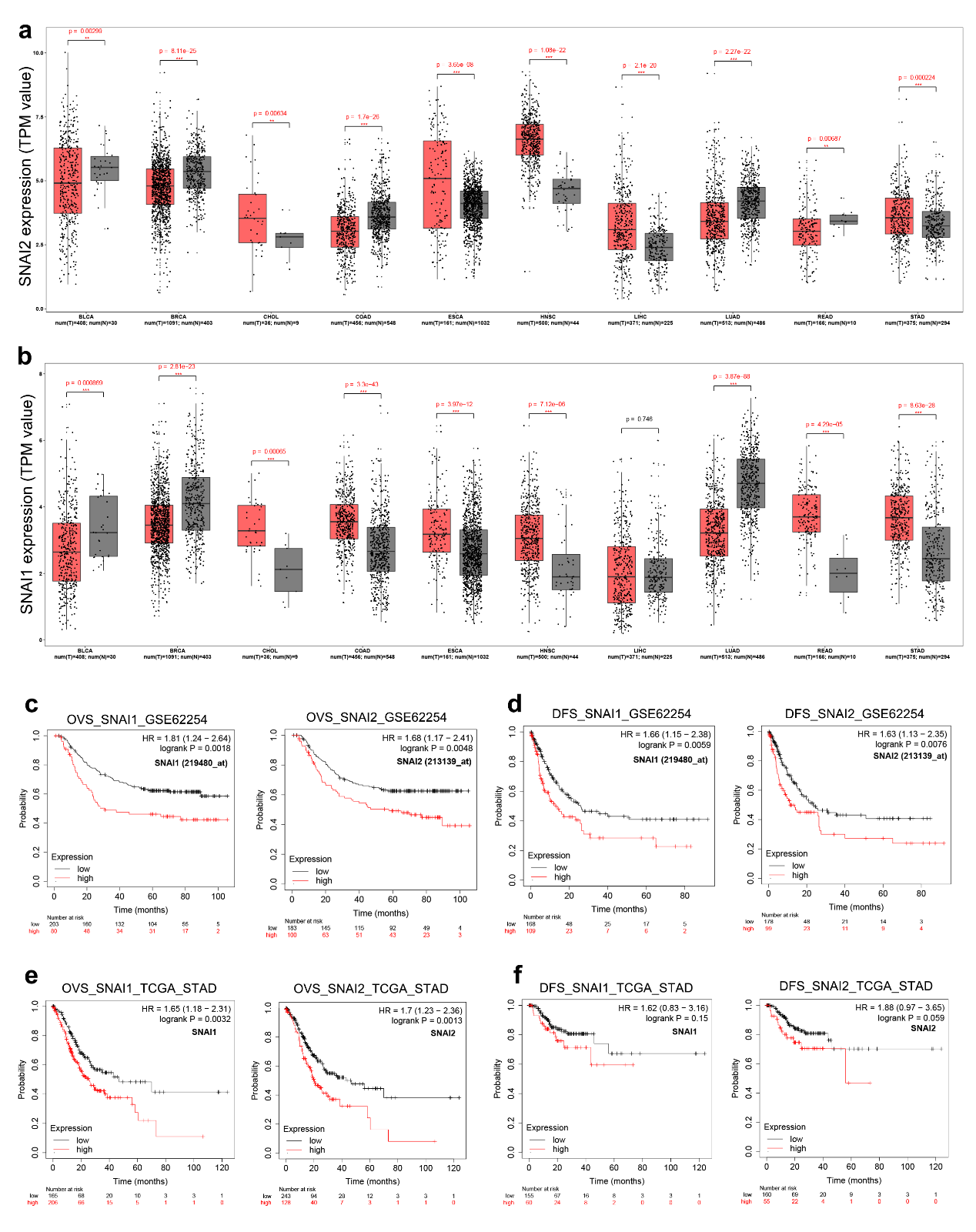


**Figure S1**

**Supplementary Figure S2:** ELF3-AS1 inhibits GC metastasis *in vitro* and *in vivo*. (A) The percentage of apoptotic cells was determined by flow cytometric analysis (left panel). The statistical results were shown on the left panel. (B, C) The cell proliferation assays were performed in the GC cells that knocked down (B) or overexpressed ELF3-AS1 (C). (D, E) The effects of ELF3-AS1 knockdown on GC cells migration were assessed by wound healing assays in GC cell lines. Scale bars=100μm. (F) The effects of ELF3-AS1 knockdown on GC cells invasion were assessed by transwell assays in two GC cell lines. Scale bars=50μm (G) The cell colony formation assay was performed in the GC cell line that overexpressed ELF3-AS1. (H, I) The wound healing assays and transwell assays were performed in HGC-27 cells to detect the effect of ELF3-AS1 overexpression on GC cells metastasis. Scale bars=100μm (J) The volumes of xenograft tumor from negative control group and the ELF3-AS1 knockdown group were measured. Scale bars=50μm. (K) The weight of xenograft tumor from negative control group and the ELF3-AS1 knockdown group were measured. **, P < 0.01.


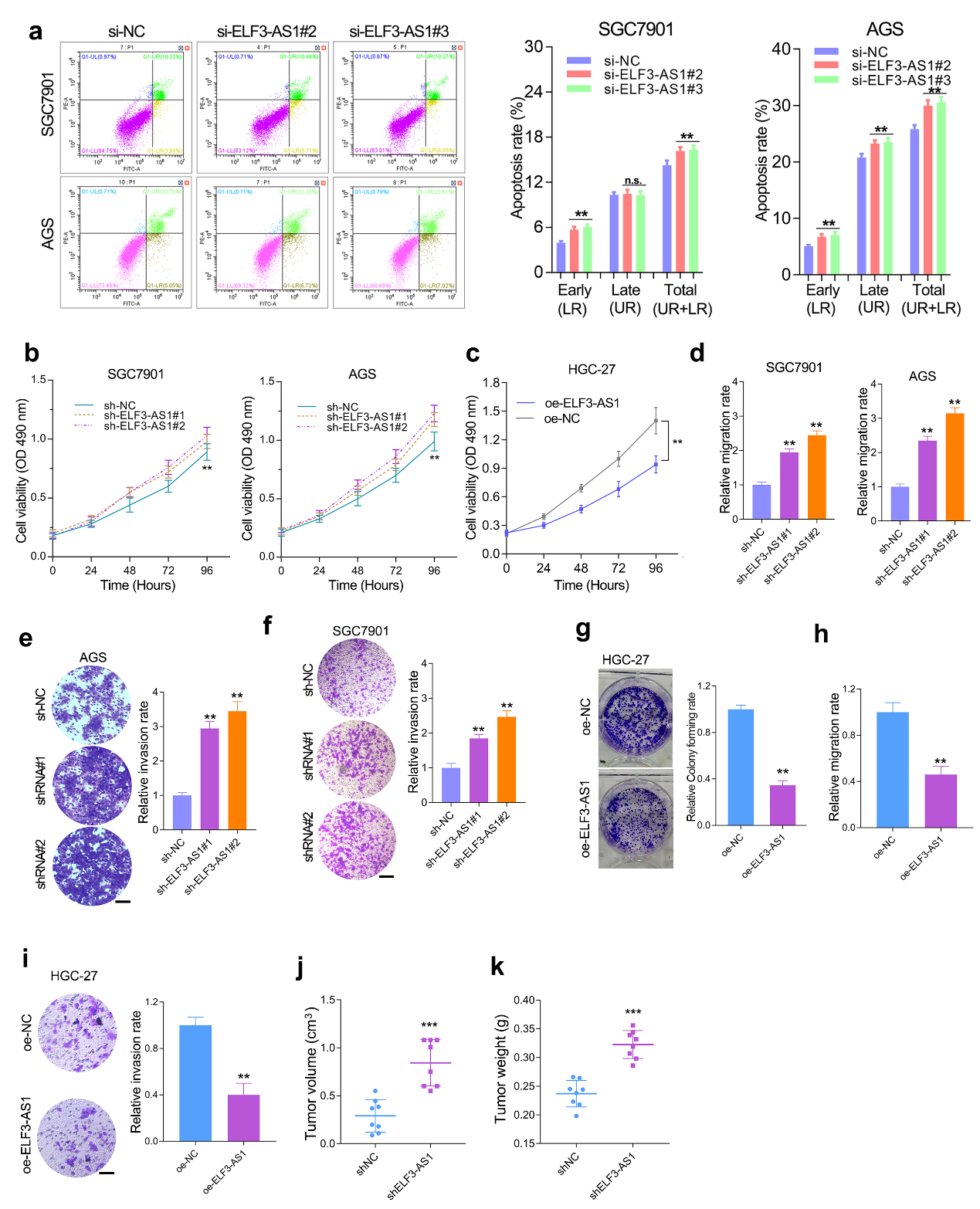


**Figure S2**

**Supplementary Figure S3:** The gene expression profile of ELF3-AS1 knockdown and SNAI2 overexpression are very similar. (A) The heat map reveals clusters of genes altered by ELF3-AS1 knockdown (left panel) and SNAI1/2 overexpression (right panel). The red color indicates genes that are up regulated compared with the control cells, and the green color indicates genes that are down regulated compared with the control cells. (B) The genes significantly regulated by both ELF3-AS1 knockdown (Log2FC>0.7) and SNAI2 overexpression were shown in the heat map. The numbers in the figure represent the expression changes (Log2FC value) after ELF3-AS1 knockdown and SNAI1/2 overexpression. (C) The Venn plot of genes significantly regulated by SNAI2 overexpression and ELF3-AS1 knockdown. (D) The validation of genes that significantly regulated by both SNAI2 and ELF3-AS1 using qPCR method. **, P < 0.01.


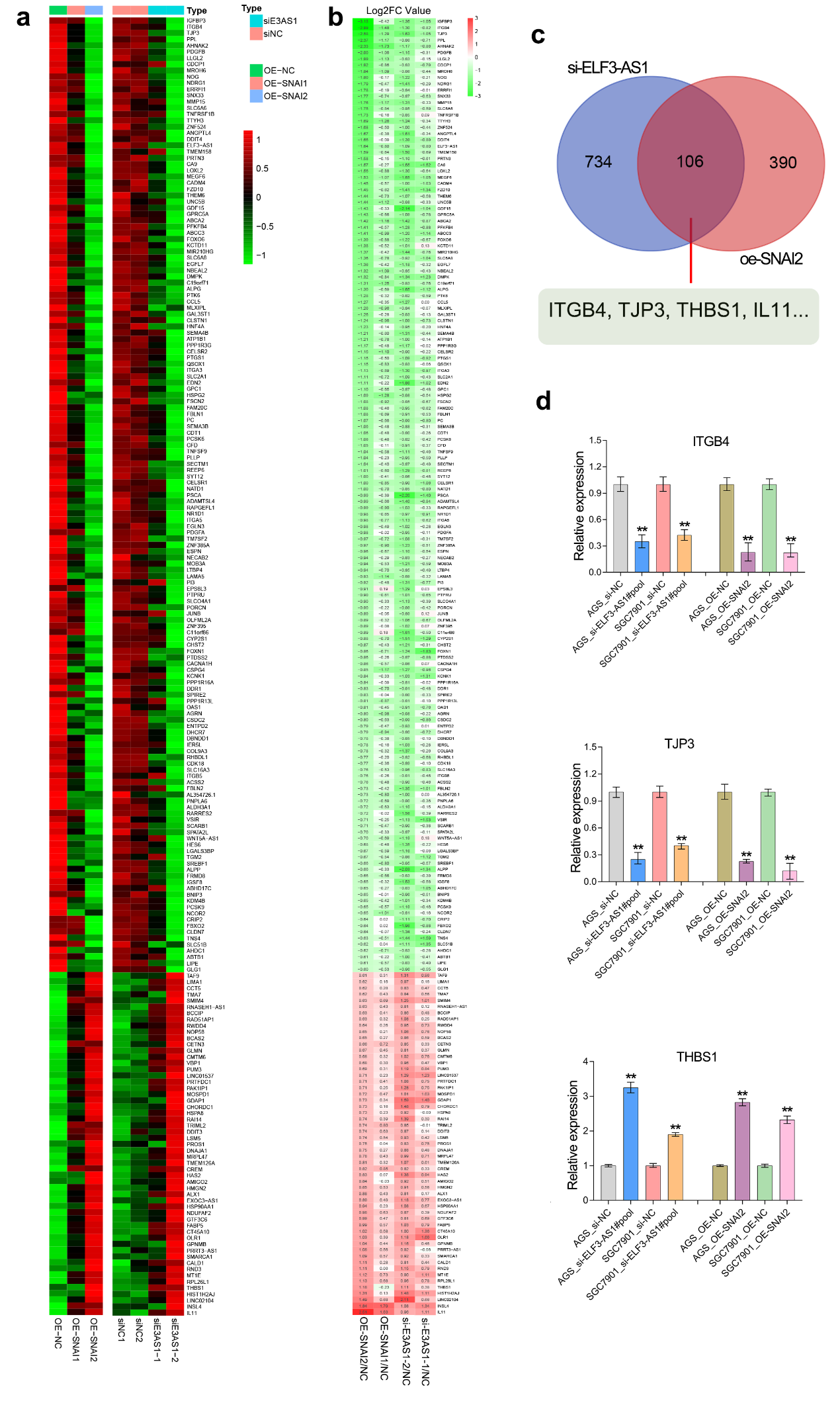


**Supplementary Figure S4:** The ILF2/ILF3 complex dynamically regulates mRNA stability of ELF3-AS1 and ELF3. (A-C) The effect of ILF3 knockdown on the expression of ELF3-AS1 and ELF3 were determined by qRT-PCR assays. (D-F) The effect of ILF2 knockdown on the expression of ELF3-AS1 and ELF3 were determined by qRT-PCR assays. (G, H) The expression of ELF3-AS1 and ELF3 were determined in the ILF2-silenced GC cell lines by qRT-PCR assays. (I) The normalized expression levels (FPKM value) of different transcripts of ELF3 and ELF3-AS1 after silencing ILF2 or ILF3 or overexpressing SNAI2 and SNAI1. **P < 0.01


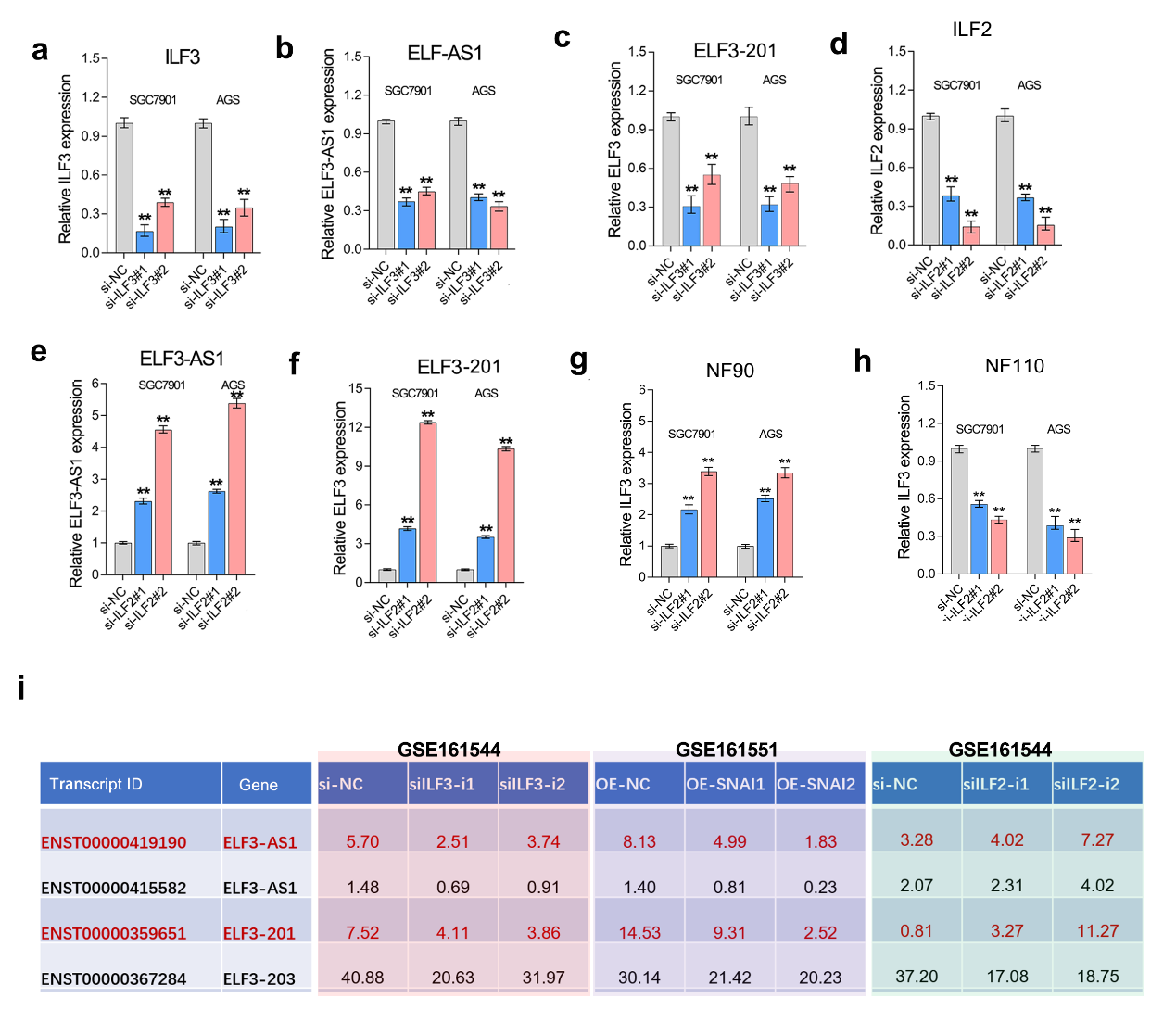


**Figure S4**

**Supplementary Figure S5:** Survival Analysis of ELF3-AS1 and SNAI2 in pan-cancer. ELF3-AS1 and SNAI2 possessed opposite prognosis in pan-cancer.


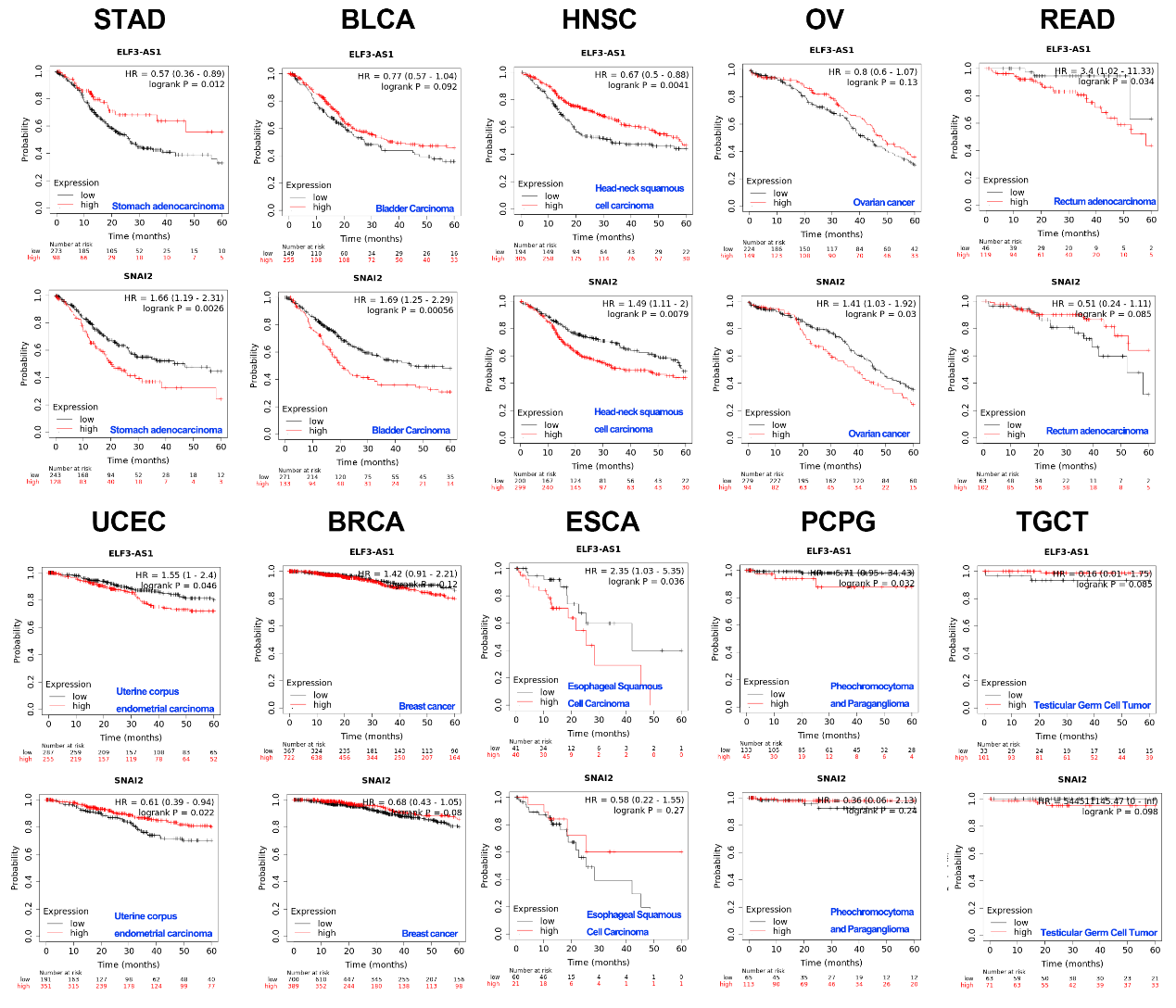


**Figure S5**
