# Supplementary material for: LncRNA ELF3-AS1 inhibits gastric cancer by forming a negative feedback loop with SNAI2 and regulates ELF3 mRNA stability via interacting with ILF2/ILF3 complex
Source: J Exp Clin Cancer Res. 2022 Dec 2;41:332. doi: 10.1186/s13046-022-02541-9 (PMC9716751; doi:10.1186/s13046-022-02541-9)
Supplement: Supplementary file 2 — Additional file 2. [file 13046_2022_2541_MOESM2_ESM.docx]

**Table S1.**

Primers and siRNAs used in this study.

| Gene Name | Sequence (5→`3`) | |
| --- | --- | --- |
| siRNA | | |
| siELF3-AS1#1 | GGCUGACCUGAGUCAGAAATT | UUUCUGACUCAGGUCAGCCTT |
| siELF3-AS1#2 | GGAGAGGAGUUACUAGGUUTT | AACCUAGUAACUCCUCUCCTT |
| siELF3-AS1#3 | GCCAGAGAAUUGGCUACAATT | UUGUAGCCAAUUCUCUGGCTT |
| siELF3#1 | GCUGCAACCUGUGAGAUUATT | UAAUCUCACAGGUUGCAGCTT |
| siELF3#2 | CCUCUGCAAUUGUGCCCUUTT | AAGGGCACAAUUGCAGAGGTT |
| siELF3#3 | CCAUGAGGUACUACUACAATT | UUGUAGUAGUACCUCAUGGTT |
| si-ILF2#1 | CUUUGUACCACAUAUCCCATT | UGGGAUAUGUGGUACAAAGTT |
| si-ILF2#2 | GAACUCCAUUUGGAUAUCATT | UGAUAUCCAAAUGGAGUUCTT |
| si-ILF3#1 | GACCGAAAUUUGCUGCUAATT | UUAGCAGCAAAUUUCGGUCTT |
| si-ILF3#2 | GGAGGUUGAUGGCAAUUCATT | UGAAUUGCCAUCAACCUCCTT |
| qRT-PCR | Forward primer | Reverse primer |
| SNAI2 | GCATTTGCAGACAGGTCAAA | TCCTCATGTTTGTGCAGGAG |
| SNAI1 | AGCGAGCTGCAGGACTCTAA | GGACAGAGTCCCAGATGAGC |
| ILF3-Total | CCCCAGAGGACGACAGTAAA | CTCCTTACACAGCAGCACCA |
| NF110 | CCTTGTCTCACCACCAACCT | CCAGAAGCTCCCAACTATGC |
| NF90 | CGGAGTCATTCTGGCTCTCT | CGCAAAATCTTGCAAGTCAA |
| ILF2 | AACAGTGCCACCCAATCTTC | CCAGGAAAACGAATCCTCAA |
| ELF3 | GAAGTGACGTGGACCTGGAT | CTTCTTGCCCTCGAGACAGT |
| ACTIN | ATCGTCCACCGCAAATGCTTCTA | AGCCATGCCAATCTCATCTTGTT |
| P21 | ttagcagcggaacaaggagt | gccgagagaaaacagtccag |
| P53 | ATGGAGGAGCCGCAGTCAGATC | CCATTGTTCAATATCGTCCGGG |
| CDK6 | AACACCCTTGGTGGCTTATG | TTTCCTTGGAGAAGCAGAGC |
| CASP7 | CACCTATCCTGCCCTCACAT | TTATGGGCCAGGCTTACATC |
| MALAT1 | AAAGCAAGGTCTCCCCACAAG | GGTCTGTGCTAGATCAAAAGGCA |
| GAPDH | TCACCAGGGCTGCTTTTA | AAGGTCATCCCTGAGCTGAA |
| ELF3-201 | ATTGTGTTTCGGGCTGAGTC | CCAGGTATGCAGGTGTGTTG |
| ELF3-AS1 | CGGCTCTGCTTGAAAGTTCT | CTGACTGAACCCAAGCCATT |
| CHIP-primer | Forward primer | Reverse primer |
| ELF3-AS1_P | tcccccatttgtctaacagg | CCTACCCACAGGTAGCCTCA |
| miRNA qPCR | Forward primer | Reverse primer |
| U6 | CTCGCTTCGGCAGCACA | AACGCTTCACGAATTTGCGT |
| miR-33a | gggGTGCATTGTAGTTGCA | CAGTGCGTGTCGTGGAGT |
| miR-33b | gggGTGCATTGCTGTTGCA | CAGTGCGTGTCGTGGAGT |
| miR-203a | gggGTGAAATGTTTAGGACC | CAGTGCGTGTCGTGGAGT |
